# Socioeconomic, Patient, and Hospital Determinants for the Utilization of Peripheral Nerve Blocks in Total Joint Arthroplasty

**DOI:** 10.1213/ANE.0000000000007107

**Published:** 2025-02-14

**Authors:** Joshua M. Bonsel, Hanish Kodali, Jashvant Poeran, Gouke J. Bonsel

**Affiliations:** From the * Department of Orthopedics and Sports Medicine, Erasmus Medical Center, Rotterdam, the Netherlands; † Department of Population Health and Policy, Icahn School of Medicine, Mount Sinai Hospital, New York, New York; ‡ EuroQol Research Foundation, Rotterdam, the Netherlands.

## Abstract

**BACKGROUND:**

While peripheral nerve blocks (PNBs) are associated with various improved outcomes in patients undergoing total hip or knee arthroplasty (THA/TKA), disparities in PNB utilization have been reported. This study assessed the importance of socioeconomic, demographic, clinical, and hospital determinants in explaining PNB utilization using the population-attributable risk (PAR) framework. Subsequently, we examined the association between PNB use and 3 secondary outcomes: Centers for Medicare and Medicaid Services (CMS)-defined complications, 90-day all-cause readmissions, and length of stay >3 days.

**METHODS:**

This retrospective cohort study included 52,926 THA and 94,795 TKA cases from the 5% 2012 to 2021 Medicare dataset. Mixed-effects logistic regression models measured the association between study variables and PNB utilization. Variables of interest were demographic (age, sex), clinical (outpatient setting, diagnosis, prior hospitalizations in the year before surgery, Deyo-Charlson index, obesity, (non)-opioid abuse, smoking), socioeconomic (neighborhood Social Deprivation Index, race and ethnicity) and hospital variables (beds, ownership, region, rurality, resident-to-bed ratio). The model was used for the calculation of variable-specific and variable category-specific PARs (presented in percentages), reflecting the proportion of variation in PNB use explained after eliminating variables (or groups of variables) of interest with all other factors held constant. Subsequently, regression models measured the association between PNB use and secondary outcomes. Associations are presented with odds ratios (ORs) and 95% confidence intervals (95% CIs).

**RESULTS:**

Socioeconomic and demographic variables accounted for only a small proportion of variation in PNB use (up to 3% and 7%, respectively). Clinical (THA: 46%; TKA: 34%) and hospital variables (THA: 31%; TKA: 22%) were the primary drivers of variation. In THA, variation by clinical variables was driven by increased PNB use in the inpatient setting (OR, 1.28 [95% CI, 1.07–1.53]) and decreased use in patients with ≥2 prior hospitalizations (OR, 0.72 [95% CI, 0.57–0.90]). Moreover, nonosteoarthritis diagnoses associated with reduced PNB utilization in THA (OR, 0.64 [95% CI, 0.58–0.72]) and TKA (OR, 0.35 [95% CI, 0.34–0.37]).

In TKA, PNB use was subsequently associated with fewer complications (OR, 0.82 [95% CI, 0.75–0.90]) and less prolonged length of stay (OR, 0.90 [95% CI, 0.86–0.95]); no association was found for readmissions (OR, 0.98 [95% CI, 0.93–1.03]). In THA, associations did not reach statistical significance.

**CONCLUSIONS:**

Among THA and TKA patients on Medicare, large variations exist in the utilization of PNBs by clinical and hospital variables, while demographic and socioeconomic variables played a limited role. Given the consistent benefits of PNBs, particularly in TKA patients, more standardized provision may be warranted to mitigate the observed variation.

**KEY POINTS**

**Question:** What is the importance of socioeconomic, demographic, clinical, and hospital determinants for the utilization of peripheral nerve blocks (PNBs) in total hip and knee arthroplasty patients (THA/TKA), and do PNBs associate with improved outcomes?

**Findings:** In both THA and TKA patients on Medicare, clinical (eg, indication for surgery) and hospital variables explained most variation in PNB use, while demographic and socioeconomic variables played a limited role; in TKA patients, PNBs were also associated with reduced complications and length of stay.

**Meaning:** Our findings emphasize substantial individual and hospital practice variation in PNB use; as PNBs are consistently associated with improved outcomes, particularly in TKA patients, the findings are a plea for more standardized provision of PNBs.

While peripheral nerve blocks (PNBs) have been associated with improved outcomes in patients undergoing total hip or knee arthroplasty (THA/TKA),^1,2^ disparities in their utilization based on patient and hospital determinants have been reported.^3–5^ Indeed, studies have shown that being younger, un(der)insured, or belonging to a minority group is associated with lower odds of receiving PNBs.^6,7^ At the hospital level, a rural location and teaching status are associated with decreased PNB utilization.^4^ However, size and even direction of effect are not always consistent as illustrated by a recent study including both patient- and hospital-level variables.^8^

Separating the effect of factors of interest is complex in both statistical analysis and daily practice, as they are often interrelated. One should account for the so-called “level” of their action (eg, patient-level versus hospital-level effects). Also, the impact indicator should reflect both the prevalence and the strength of the determinants. For example, even if there is a very strong association indicating Black patients receive fewer PNBs, its population-level impact will be limited in a hypothetical population with only a few Black patients. In a population with more Black patients, the population-level impact may still be limited if the strength of the race-PNB association is weaker than other or higher-level factors. The population-attributable risk (PAR) concept, combined with stepwise analysis methods to account for the aforementioned “level” of action issue, provides a valuable approach for this purpose.^9,10^ The PAR assesses the impact of a determinant (or study variable of effect) in terms of the proportion of PNB use accounted for by that determinant.

Our study aimed to get a deeper understanding of the source of PNB variation. We estimated the importance of the socioeconomic background (including race/ethnicity and a proxy for socioeconomic status [SES]), demographic, clinical, and hospital determinants of the patient in explaining PNB utilization. We hypothesized a greater role of hospital versus patient variables and that within the latter PNB use would be lower in minority patients and those with a lower SES. We subsequently examined the association between PNB use and 3 important outcomes related to THA/TKA (complications, 90-day all-cause readmissions, and length of stay) hypothesizing that PNB would be associated with improved outcomes, further emphasizing the importance of minimizing the hypothesized variation in PNB use.

## METHODS

### Data

In this retrospective cohort study, we analyzed inpatient and outpatient THAs and TKAs performed between 2012 and 2021 (all data available to our research group) as recorded in the Medicare Limited Dataset.^11^ Given the deidentified nature of the data source, this study was exempt from full review by the Mount Sinai Institutional Review Board (STUDY-20-01677). This study followed the Strengthening the Reporting of Observational Studies in Epidemiology (STROBE) guidelines.^12^

**Figure.**
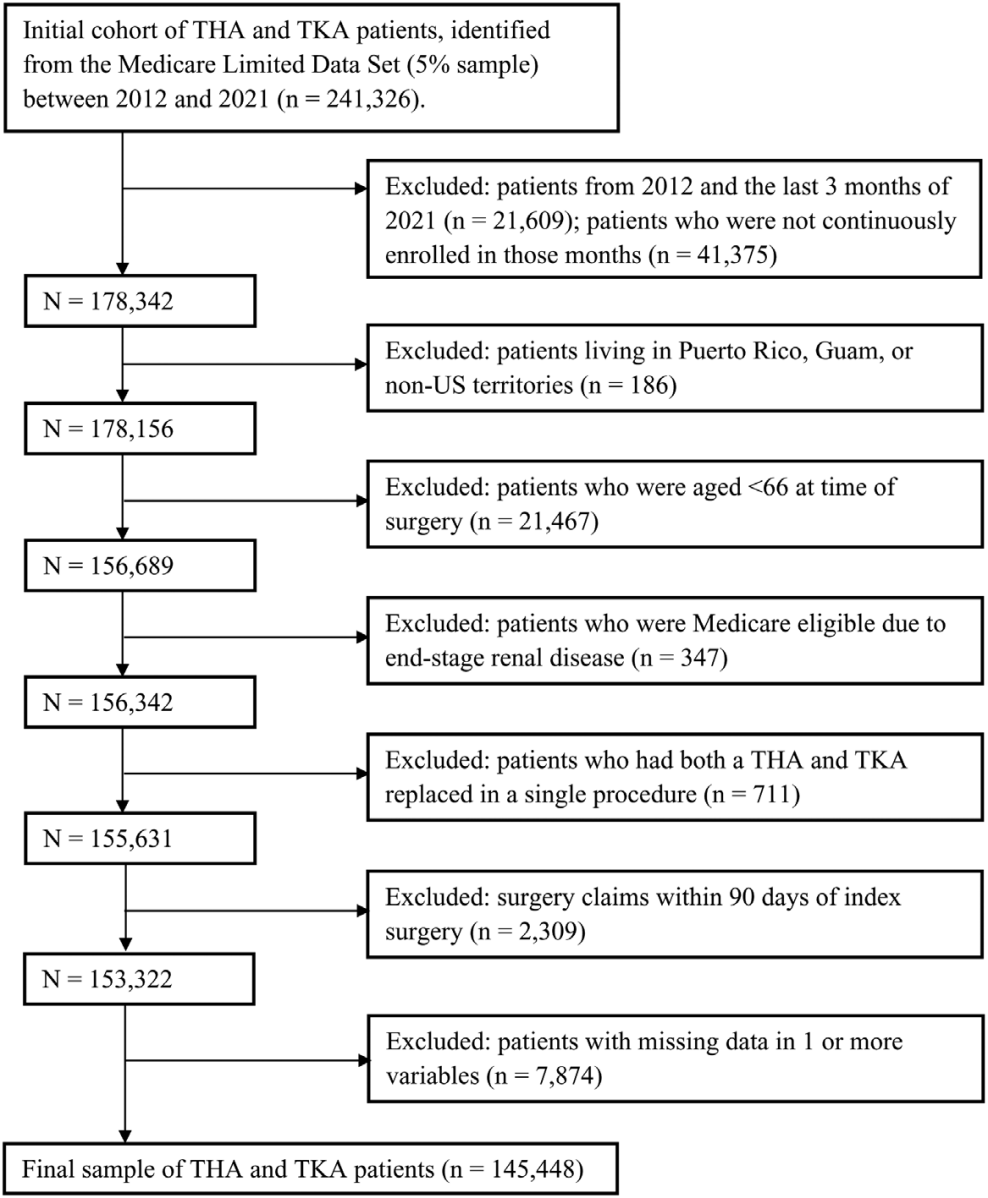
Flowchart depicting sample selection.

The Medicare database includes patient-level claims of all Medicare-insured patients in the United States. The inpatient and outpatient files contain an incomplete overview of the PNBs used; therefore, we also used the Carrier file to define PNBs, which made it necessary to use the 5% random sample. Each encounter contains information on procedural (Current Procedural Terminology [CPT]) and diagnosis-related (International Classification of Diseases ninth Revision codes [ICD]) codes. In 2015 the International Classification of Diseases tenth Revision coding system (ICD-10) was introduced. As this database mainly captures individuals aged over 65 and/or with disabilities, younger patients with or without private insurance are not included.

### Inclusion and Exclusion Criteria of the Sample

We constructed an initial cohort of 241,326 primary THA (CPT: 27130, ICD-9: 81.51, ICD-10: 0SR90, 0SRB0) and TKA (CPT: 27477, ICD-9: 81.54, ICD-10: 0SRC0, 0SRD0) patients. To define comorbidity prevalence and complication rate before and after surgery, respectively, we excluded patients who had surgery in 2012 or the last 3 months of 2021 (n = 21,609). Subsequently, we excluded patients who were not continuously enrolled in the database for at least 1 year prior to and 3 months after their joint arthroplasty (n = 41,375); patients inhabiting unincorporated territories of the United States (n = 186); aged <66 (n = 21,467); eligibility for Medicare due to end-stage renal disease (n = 347); patients having claims of both THA and TKA simultaneously (n = 711). For each patient, we only kept index arthroplasties which were 90 days apart, excluding another n = 2309 claims. Therefore, repeat procedures are not expected.^13^ Patients may have had a contralateral procedure or procedure of another joint. Given the nature of the data, we were unable to determine whether patients had prior primary joint replacement before enrolling in Medicare. This process resulted in a sample of 153,322 patients (Figure).

### Variables

Variables were selected based on the potential influence on PNB use either found in previous studies or based on clinical judgement.^4–8,14^ Directly obtained were age, sex, and year of surgery. Race and ethnicity was grouped as White, Black, and other (Asian, Hispanic, North American Native, other).^15^ Diagnosis was defined as either osteoarthritis or nonosteoarthritis using ICD codes (attached in Supplemental Digital Content 1, Supplemental Table 1, http://links.lww.com/AA/E911). We calculated the Deyo-Charlon comorbidity index and categorized it into 0, 1, 2, and ≥3.^16,17^ We identified a history of obesity, smoking, nonopioid, and opioid abuse. Prior hospitalization was defined as any acute care hospitalization in the 365 days before receiving surgery (excluding index arthroplasties) and was categorized into 0, 1, or ≥2. The effect of these variables on arthroplasty outcomes is well known, however, to our knowledge they have not been studied in the context of PNB utilization.

The 2019 neighborhood Social Deprivation Index (SDI) provides details on the place of living and was individually linked to the state-county ID. The SDI is a composite measure based on 7 characteristics, where a 1 to 100 SDI score is calculated using 5-year (2014–2019) averaged data from the American Community Survey: % living in poverty, % with <12 years of education, % single-parent households, % living in rented housing units, % living in the overcrowded housing unit, % of households without a car, and % nonemployed adults under 65 years of age.^18^ We categorized it into 5 groups based on the thresholds for the 20th, 40th, 60th, and 80th percentiles.^19^ Neighborhood deprivation indices are generally stable over time, and the currently applied measure is assumed valid for the included timeframe.^20^

The following hospital data were derived from the 2017 Hospital Inpatient Prospective Payment System 2017 impact file: beds (0–150, 150–499, and ≥500), ownership (government, physician/proprietary, voluntary), rurality (large urban, small urban and rural), region (Northeast, South, Midwest, and West) and resident-to-bed ratio.^21^ The resident-to-bed ratio is defined as the ratio of (interns + residents)/average operating beds; this ratio has previously been used as a proxy for teaching intensity. The ratio was categorized as <0.05 reflecting no teaching, 0.05 to 0.249 reflecting minor teaching, and ≥0.25 reflecting major teaching.^22,23^ Slight variations in hospital variables may occur over time: to maintain the ability to estimate the effects of these variables at the hospital level, we linked the IPPS file available in the middle of the included timeframe.

Missingness in any of the variables was present in 5% of THA and TKA patients and was mainly attributable to hospitals not being recorded in the IPPS file. Missingness was unrelated to outcomes; therefore, we conducted a complete case analysis (Supplemental Digital Content 2, Supplemental Figures 1–4, http://links.lww.com/AA/E912). This resulted in a final sample of n = 147,721 patients.

### Peripheral Nerve Block Utilization, Complication Incidence, and Length of Stay (Outcomes)

Our primary outcome was the utilization of any form of PNB for the received joint arthroplasty, which was defined using CPT and ICD codes (Supplemental Digital Content 1, Supplemental Table 1, http://links.lww.com/AA/E911) submitted on the day of surgery, or at maximum 1 day before or 1 day after surgery. The 3 secondary outcomes of interest were Centers for Medicare and Medicaid Services (CMS)-defined complication (definition in Supplemental Digital Content 3, Supplemental Table 2, http://links.lww.com/AA/E913), 90-day all-cause readmissions and length of stay >3 days. Based on empirical evidence, CMS has defined outcome measures for commonly performed procedures, which are used to determine hospitals’ performance and to adjust reimbursement rates. The measure captures negative outcomes that are likely attributable to the studied procedure, and which represent the quality of the provided care. In THA and TKA, a CMS-defined complication includes acute myocardial infarction, pneumonia, or sepsis/septicemia/shock during the admission or within 7 days from the date of admission; pulmonary embolism, surgical site bleeding or death during the admission or within 30 days from the date of admission; mechanical complications, surgical site bleeding or peri-prosthetic joint/wound infection during the admission or within 90 days from the date of admission.^24^

### Statistical Analysis

THA and TKA patients were analyzed separately. Patient and hospital variables were compared between recipients and nonrecipients of a PNB. We used χ2 test for categorical variables.

For our first research question, we aimed to explore the relative contribution of each variable to the use of PNBs whilst accounting for potential confounding effects. Because hospital-level variables generally strongly affect PNB use, unmeasured factors may play a role. Logistic regression null models were compared with and without a random intercept for the hospital. Model fit improved drastically, with intraclass correlation coefficients (ICC) of 0.67 (THA) and 0.47 (TKA). The addition of a random intercept for the patient (3-level model) to account for contralateral procedures had minimal effect and was therefore not included in the final models. All variables of interest were entered as fixed effects: age, sex, outpatient setting, diagnosis, prior hospitalizations, Deyo index, obesity, (non)-opiod abuse, smoking, SDI, race and ethnicity, hospital beds, ownership, region, rurality, and resident-to-bed ratio. We assessed potential multicollinearity among variables using Spearman rank correlation indices. With all pairwise correlation indices <0.4, we determined the risk of multicollinearity to be low, as commonly accepted thresholds range from 0.5 to 0.8.^25,26^ The year of the surgery was adjusted for in the analysis, but was not considered a variable of interest with regard to our primary research question; it would have provided no opportunity for practice change. Within a categorical variable, the category with the highest amount of PNBs used was selected as a reference category. The reference category was generally the same for THA and TKA; if not, we opted to keep the same category as a reference in both THA and TKA.

To visualize the contribution of each variable to the use of PNBs we calculated PARs. Conventionally, PARs are calculated as [Pr(O) – Pr(O|E)]/ Pr(O), where Pr(O) is the probability of outcome in the study population, Pr(O|E) is the hypothetical probability of outcome if the variable of interest were eliminated.^27^ This approach fails to take confounders into account; therefore, we used abovementioned regression models to estimate confounder-adjusted PARs, currently the most efficient method available.^27^ We followed a previously described approach.^9^ Study population estimates of the number of PNBs used [Pr(O)] and estimates for each variable’s category predicting the least amount of PNBs [Pr(O|E)] were obtained using the regression models. The PAR for each variable was calculated using the abovementioned formula (univariable PARs). The interpretation of an univariable PAR is the maximum % of PNB variation explained by eliminating that variable while controlling for confounding effects of other variables, assuming all potential confounders have been accounted for.

Sequential PARs were calculated for groups of variables: demographic (age, sex), clinical (outpatient setting, diagnosis, prior hospitalizations, Deyo index, obesity, (non)-opioid abuse, smoking), socioeconomic (SDI, race and ethnicity) and hospital (hospital beds, ownership, region, rurality, resident-to-bed ratio) variables. First, estimates for a group of variables set to the least amount of PNBs are obtained, and the PAR is calculated. Subsequently, the next group of variables is also set to the least amount of PNBs and new estimates are obtained, and the difference in PAR is calculated. This second procedure is repeated until all 4 groups are eliminated. The sequential PAR at each step represents the added variation explained by a group of variables after the preceding group(s) of variables have been eliminated. The order of eliminating groups of variables influences the sequential PARs because variables are potentially interrelated.^28,29^ Our primary order of interest was demographic > clinical > socioeconomic > hospital-level variables, based on the assumption that biological variables precede hospital variables. Reverse ordering was also assessed to evaluate the degree of interrelatedness of variables. If PAR estimates are similar for different orderings, groups of variables act independently on the utilization of PNBs. If PAR estimates differ, variable groups are at least statistically interrelated; for example, eliminating clinical variables may explain 10% of the variation in PNB. However, by eliminating demographics before clinical variables, the additional effect of clinical variables may be reduced to 5%, which would indicate interrelatedness between these groups of variables. In case of interrelatedness, univariable PARs exceed the total PAR for all risk factors combined.^30^

For our second research question, mixed-effects logistic regression models with adjustment for the same variables measured the association between PNBs and the 3 secondary outcomes.

Two sensitivity analyses were conducted. Firstly, we checked whether there was a variation in PNB utilization by county of residence beyond the variation measured with the SDI. We re-ran the primary models replacing SDI by state-county ID (n = 3200) as random intercept (3-level model), and entering both simultaneously, and evaluated coefficients and model fit. Secondly, dual eligibility status (Medicare and Medicaid) was added as a variable of interest. As this variable has been collected since 2017,^31^ all models were re-run from using data from 2017 onwards.

**Table 1.** Descriptive Statistics of Total Hip and Knee Arthroplasty Patients by the Utilization of Any Type of PNB

	Total hip arthroplasty			Total knee arthroplasty		
Variables	No PNB	PNB	*P*-value	No PNB	PNB	*P*-value
n	47,914	4086		39,989	53,459	
Patient characteristics						
Age			.085			<.001
65–69	10,774 (22.5)	932 (22.8)		10,254 (25.6)	12,871 (24.1)	
70–74	14,572 (30.4)	1243 (30.4)		13,264 (33.2)	17,842 (33.4)	
75–79	11,034 (23.0)	998 (24.4)		9370 (23.4)	13,404 (25.1)	
80–84	7051 (14.7)	564 (13.8)		4956 (12.4)	6786 (12.7)	
>84	4483 (9.4)	349 (8.5)		2145 (5.4)	2556 (4.8)	
Female	30,186 (63.0)	2622 (64.2)	.141	25,670 (64.2)	34,046 (63.7)	.112
Inpatient (vs outpatient)	43,726 (91.3)	3685 (90.2)	.022	35,562 (88.9)	44,193 (82.7)	<.001
Diagnosis nonosteoarthritis	11,904 (24.8)	841 (20.6)	<.001	9257 (23.1)	6910 (12.9)	<.001
Prior hospitalizations			.027			<.001
0	39,949 (83.4)	3434 (84.0)		34,611 (86.6)	46,984 (87.9)	
1	5889 (12.3)	511 (12.5)		4162 (10.4)	5200 (9.7)	
≥2	2076 (4.3)	141 (3.5)		1216 (3.0)	1275 (2.4)	
Deyo index			.834			.835
0 (healthiest)	17,600 (36.7)	1491 (36.5)		14,449 (36.1)	19,177 (35.9)	
1	10,531 (22.0)	905 (22.1)		9512 (23.8)	12,747 (23.8)	
2	7668 (16.0)	637 (15.6)		6291 (15.7)	8408 (15.7)	
≥3 (least healthy)	12,115 (25.3)	1053 (25.8)		9737 (24.3)	13,127 (24.6)	
Obese	11,132 (23.2)	958 (23.4)	.772	12,138 (30.4)	16,888 (31.6)	<.001
Abuse of nonopioids	2880 (6.0)	268 (6.6)	.169	1485 (3.7)	2221 (4.2)	.001
Abuse of opioids	460 (1.0)	58 (1.4)	.006	270 (0.7)	387 (0.7)	.399
Smoking	4910 (10.2)	396 (9,7)	.271	3892 (9.7)	4460 (8.3)	<.001
SDI score			<.001			<.001
Q1 (most affluent)	10,083 (21.0)	789 (19.3)		7598 (19.0)	10,977 (20.5)	
Q2–Q4	28,854 (60.2)	2360 (57.8)		23,829 (59.6)	32,003 (59.9)	
Q5 (least affluent)	8977 (18.7)	937 (22.9)		8562 (21.4)	10,479 (19.6)	
Race and ethnicity			.016			.001
White	45,255 (94.5)	3832 (93.8)		36,592 (91.5)	49,276 (92.2)	
Black	1878 (3.9)	163 (4.0)		2040 (5.1)	2461 (4.6)	
Asian, Hispanic, North American native, other	781 (1.6)	91 (2.2)		1357 (3.4)	1722 (3.2)	
Year of surgery			<.001			<.001
2013	4566 (9.5)	341 (8.3)		4673 (11.7)	5454 (10.2)	
2014	4690 (9.8)	384 (9.4)		4976 (12.4)	4994 (9.3)	
2015	5396 (11.3)	361 (8.8)		5236 (13.1)	5219 (9.8)	
2016	5748 (12.0)	378 (9.3)		5576 (13.9)	5941 (11.1)	
2017	5884 (12.3)	464 (11.4)		4946 (12.4)	6787 (12.7)	
2018	6029 (12.6)	547 (13.4)		4565 (11.4)	7165 (13.4)	
2019	6218 (13.0)	620 (15.2)		4446 (11.1)	7424 (13.9)	
2020	5334 (11.1)	519 (12.7)		3202 (8.0)	5730 (10.7)	
2021	4049 (8.5)	472 (11.6)		2369 (5.9)	4745 (8.9)	
Hospital characteristics						
Hospital beds			<.001			<.001
0–150	14,627 (30.5)	1230 (30.1)		10,815 (27.0)	15,629 (29.2)	
150–499	25,639 (53.5)	2086 (51.1)		21,867 (54.7)	27,345 (51.2)	
≥500	7648 (16.0)	770 (18.8)		7307 (18.3)	10,485 (19.6)	
Hospital ownership			<.001			.024
Government	4950 (10.3)	521 (12.8)		4274 (10.7)	5568 (10.4)	
Physician/proprietary	7499 (15.7)	976 (23.9)		7349 (18.4)	10,179 (19.0)	
Voluntary	35,465 (74.0)	2589 (63.4)		28,366 (70.9)	37,712 (70.5)	
Region			<.001			<.001
Northeast	9098 (19.0)	539 (13.2)		6334 (15.8)	8954 (16.7)	
South	17,649 (36.8)	1505 (36.8)		16,671 (41.7)	20,975 (39.2)	
Midwest	11,561 (24.1)	1230 (30.1)		9512 (23.8)	13,932 (26.1)	
West	9606 (20.0)	812 (19.9)		7472 (18.7)	9598 (18.0)	
Rurality			<.001			<.001
Large urban	23,333 (48.7)	2294 (56.1)		17,409 (43.5)	25,815 (48.3)	
Small urban	20,681 (43.2)	1457 (35.7)		18,405 (46.0)	22,799 (42.6)	
Rural	3900 (8.1)	335 (8.2)		4175 (10.4)	4845 (9.1)	
Resident-to-bed ratio			.249			<.001
No teaching	30,173 (63.0)	2525 (61.8)		26,866 (67.2)	36,093 (67.5)	
Minor teaching	10,861 (22.7)	941 (23.0)		9139 (22.9)	11,035 (20.6)	
Major teaching	6880 (14.4)	620 (15.2)		3984 (10.0)	6331 (11.8)	

Values are presented as n (%). *P*-values indicate differences between PNB and no PNB patients.

Abbreviations: PNB, peripheral nerve block; SDI, Social Deprivation Index.

**Table 2.** Mixed-Effects Logistic Regression Models of Patient and Hospital Variables on the Use of PNBs

	Total hip arthroplasty		Total knee arthroplasty	
	OR (95% CI)	*P*-value	OR (95% CI)	*P*-value
Intercept	0.21 (0.10–0.43)	<.001	6.92 (4.57–10.46)	<.001
Age				
65–69	0.97 (0.86–1.10)	.644	0.84 (0.80–0.88)	<.001
70–74	0.91 (0.82–1.02)	.109	0.90 (0.86–0.94)	<.001
75–79	Ref		Ref	
80–84	0.86 (0.75–0.98)	.027	0.97 (0.91–1.02)	.246
>84	0.84 (0.72–0.99)	.038	0.81 (0.75–0.88)	<.001
Female	1.02 (0.93–1.11)	.724	0.95 (0.92–0.99)	.008
Inpatient (vs outpatient)	1.28 (1.07–1.53)	.007	0.71 (0.67–0.76)	<.001
Diagnosis nonosteoarthritis	0.64 (0.58–0.72)	<.001	0.35 (0.34–0.37)	<.001
Prior hospitalizations				
0	Ref		Ref	
1	0.97 (0.86–1.10)	.653	0.97 (0.92–1.03)	.328
≥2	0.72 (0.57–0.90)	.004	0.76 (0.68–0.84)	<.001
Deyo index				
0 (healthiest)	0.96 (0.86–1.08)	.525	0.95 (0.90–0.99)	.019
1	1.00 (0.88–1.12)	.953	1.01 (0.96–1.06)	.705
2	0.93 (0.81–1.06)	.249	0.96 (0.91–1.02)	.178
≥3 (least healthy)	Ref		Ref	
No obesity	1.01 (0.91–1.11)	.868	0.98 (0.95–1.02)	.344
No abuse of nonopioids	0.96 (0.81–1.14)	.654	0.98 (0.89–1.07)	.595
No abuse of opioids	0.58 (0.40–0.84)	.004	1.04 (0.85–1.28)	.671
No smoking	1.07 (0.92–1.25)	.380	0.88 (0.83–0.94)	<.001
SDI score				
Q1 (most affluent)	Ref		Ref	
Q2–Q4	0.94 (0.82–1.07)	.328	1.00 (0.95–1.06)	.942
Q5 (least affluent)	0.98 (0.83–1.16)	.842	0.96 (0.89–1.03)	.248
Race and ethnicity				
White	Ref		Ref	
Black	0.95 (0.76–1.17)	.620	0.91 (0.84–0.99)	.029
Asian, Hispanic, North American Native, other	1.18 (0.87–1.59)	.285	0.94 (0.85–1.04)	.213
Year of surgery				
2013	0.49 (0.39–0.60)	<.001	0.83 (0.76–0.91)	<.001
2014	0.53 (0.43–0.65)	<.001	0.62 (0.56–0.68)	<.001
2015	0.42 (0.34–0.52)	<.001	0.55 (0.50–0.60)	<.001
2016	0.34 (0.28–0.42)	<.001	0.47 (0.43–0.51)	<.001
2017	0.44 (0.36–0.54)	<.001	0.68 (0.63–0.75)	<.001
2018	0.52 (0.43–0.63)	<.001	0.80 (0.74–0.87)	<.001
2019	0.64 (0.53–0.77)	<.001	0.85 (0.79–0.93)	<.001
2020	0.67 (0.56–0.79)	<.001	0.91 (0.83–0.99)	.024
2021	Ref		Ref	
Hospital beds				
≥500	Ref		Ref	
150–499	0.82 (0.60–1.13)	.219	0.85 (0.70–1.02)	.075
0–150	1.08 (0.73–1.61)	.696	0.98 (0.79–1.23)	.876
Hospital ownership				
Government	0.71 (0.45–1.13)	.152	0.94 (0.72–1.23)	.661
Physician/proprietary	Ref		Ref	
Voluntary	0.51 (0.36–0.71)	<.001	1.03 (0.85–1.26)	.732
Region				
Northeast	0.40 (0.26–0.62)	<.001	0.74 (0.58–0.94)	.012
South	0.87 (0.62–1.23)	.422	0.74 (0.61–0.90)	.003
Midwest	Ref		Ref	
West	1.09 (0.73–1.60)	.682	0.71 (0.56–0.89)	.003
Rurality				
Large urban	Ref		Ref	
Small urban	0.54 (0.40–0.72)	<.001	0.80 (0.67–0.94)	.007
Rural	0.74 (0.49–1.12)	.159	0.50 (0.40–0.63)	<.001
Resident-to-bed ratio				
No teaching	0.55 (0.35–0.86)	.010	0.96 (0.73–1.26)	.780
Minor teaching	0.62 (0.37–1.04)	.071	0.84 (0.62–1.15)	.282
Major teaching	Ref		Ref	
C-statistic	0.94		0.87	
ICC	0.67		0.49	

A random intercept was included for the hospital. Reference categories are those with the highest number of PNBs as observed in the univariable analysis. This is not always the same for hip and knee arthroplasty patients; we opted to choose the same reference category in those instances for comprehensibility. The c-statistic measured the overall model performance, and the ICC depicted the variance explained by the random intercept for the hospital.

Abbreviations: CI, confidence interval; ICC, intraclass correlation coefficient; OR, odds ratio; PNB, peripheral nerve block; SDI, Social Deprivation Index.

No post hoc power calculations were performed given the exploratory nature of this study. Interaction between independent variables was not estimated, and the standard errors obtained from the models did not account for potential clustering. We report odds ratios (ORs) with 95% confidence intervals (95% CIs), while PARs are reported in %. Model performance was assessed using the c-statistic. ICCs reflect the variance captured by the random intercept for the hospital in each model. Although a *P*-value <0.05 was considered statistically significant, they were interpreted in combination with the strength of association. We conducted our analyses using R (version 4.2.3).

## RESULTS

### Descriptive Analysis

We included 52,000 THAs and 93,448 TKAs with 7.9% (n = 4086) and 57.2% (n = 53,459) PNB use, respectively. The use of PNBs increased over the years. Univariable comparison (Table 1) of variables according to PNB use produced a similar pattern compared to the multivariable analysis (Table 2) and is therefore not separately discussed; the few exceptions to this pattern are mentioned.

### Association of Patient and Hospital Variables with Utilization of PNBs

#### THA

After adjustment, race/ethnicity and SDI did not significantly influence the utilization of PNBs. Lower odds for PNBs were seen for diagnoses other than osteoarthritis (OR, 0.64 [95% CI, 0.58–0.72]) and ≥2 prior hospitalizations (OR, 0.72 [95% CI, 0.57–0.90]). Contrary to the unadjusted analysis, an inpatient (vs outpatient) setting showed higher odds for PNB use (OR, 1.28 [95% CI, 1.07–1.53]). Stronger effect estimates were observed for hospital-level variables: voluntary (OR, 0.51 [95% CI, 0.36–0.71]) and government-owned hospitals (OR, 0.71 [95% CI, 0.45–0.13]) showed lower odds for receiving PNBs compared to physician/proprietary-owned hospitals. Patients undergoing surgery in nonteaching hospitals also had lower odds of receiving a PNB (OR, 0.55 [95% CI, 0.35–0.86]). Regional differences were substantial: patients inhabiting the West and Midwest had higher odds of receiving PNBs, as had large urban hospitals.

#### TKA

SDI did not significantly affect PNB utilization, while Black (compared to White) patients had slightly lower odds (OR, 0.91 [95% CI, 0.84–0.99]). The inpatient setting showed lower odds for PNB use (OR, 0.71 [95% CI, 0.67–0.76]). Similar to THA patients, diagnoses other than osteoarthritis (OR, 0.35 [95% CI, 0.33–0.37]) and ≥2 prior hospitalizations (OR, 0.76 [95% CI, 0.68–0.84]) were associated with lower odds of receiving PNBs. Regarding hospital variables, only region and rurality reached significance, of which associations aligned with THA results.

### Population Attributable Risks for Utilization of PNB

The highest univariable PAR of patient variables was that of having a diagnosis other than osteoarthritis (Table 3). In other words, if all THAs and TKAs were theoretically done for only nonosteoarthritis indications, this would result in 19% and 24% lower PNB utilization, respectively. In THA, the outpatient setting (13%) and ≥2 prior hospitalizations (18%) also played a large role. In THA, and to a lesser extent in TKA patients, hospital variables had high univariable PARs. In THA the largest PAR was observed for the region (38%), rurality (20%), and hospital ownership (10%). In TKA, these were rurality (14%), region (3%), and teaching status (3%).

**Table 3.** Population Attributable Risks for the Utilization of PNBs

	Total hip arthroplasty			Total knee arthroplasty		
Variable	Worst category	Predicted PNBs	Percentage	Worst category	Predicted PNBs	Percentage
Predicted blocks (unadjusted variables)^a^		3903			53,465	
Univariable PAR						
Demographics						
Age	>84	3671	6	>84	51,849	3
Sex	Male	3880	1		53,219	0
Clinical						
Inpatient (vs outpatient)	Outpatient	3395	13	Inpatient	52,788	1
Diagnosis	Nonosteoarthritis	3151	19	Nonosteoarthritis	40,732	24
Prior hospitalizations	≥2	3194	18	≥2	49,598	7
Deyo index	2	3783	3	0	53,006	1
Obesity		3908	0		53,383	0
Abuse of nonopioids		3897	0		53,452	0
Abuse of opioids		3888	0		53,469	0
Smoking	Yes	3758	4		53,298	0
Socioeconomic						
SDI score	Q2–Q4 (medium)	3847	1	Q5 (worst)	52,968	1
Race and ethnicity	Black	3771	3	Black	52,266	2
Hospital variables						
Hospital beds	150–500	3647	7	150–500	52,394	2
Hospital ownership	Voluntary	3498	10	Government	52,343	2
Region	Northeast	2422	38	West	51,821	3
Rurality	Small urban	3131	20	Rural	45,779	14
Resident-to-bed ratio	No teaching	3627	7	Minor teaching	51,923	3
Sequential PARs						
Demographics		3649	7		51,600	3
Clinical		1842	46		33,306	34
SES		1736	3		31,578	3
Hospital variables		520	31		19,615	22
Total			87			62
Sequential PARs; reverse order						
Hospital variables		1386	64		40,170	25
SES		1304	2		38,410	3
Clinical		571	19		21,175	32
Demographics		520	1		19,615	3
Total			86			63

Predicted blocks and respective PARs reflect the number of PNBs utilized if a variable is set to the worst category. In other words, the PAR reflects how much PNBs are attributable to that variable, when all other variables are kept constant. We used a mixed-effects logistic regression model to calculate the PARs with a random intercept for the hospital.

Abbreviations: PAR, population attributable risk; PNB, peripheral nerve block; SDI, Social Deprivation Index; SES, socioeconomic status; THA, total hip arthroplasty.

a The predicted number of PNBs in the THA cohort does not entirely match the observed number of PNBs (4086), while it is close for the total knee arthroplasty cohort (53,459). This is presumably due to the relatively lower incidence of PNBs applied in the THA cohort, resulting in a slightly poorer predictive capability of the model.

**Table 4.** Mixed-Effects Logistic Regression Models of PNBs on Outcomes

	Total hip arthroplasty					Total knee arthroplasty			
	OR (95% CI)	*P*-value	C-statistic	ICC	OR (95% CI)		*P*-value	C-statistic	ICC
CMS complications	0.92 (0.78–1.10)	.357	0.71	0.04	0.82 (0.75–0.90)		<.001	0.70	0.04
90-day all-cause readmissions	0.98 (0.87–1.10)	.738	0.67	0.01	0.98 (0.93–1.03)		.380	0.67	0.02
Length of stay >3 d	0.99 (0.89–1.11)	.904	0.83	0.13	0.90 (0.86–0.95)		<.001	0.78	0.14

The OR reflects the use of PNBs versus no PNB. The same set of variables that were used in the analysis of the utilization of PNBs was entered as fixed effects. A random effect for the hospital is included in the models. Length of stay is only analyzed in patients who had inpatient surgery (approximately 90% of patients). The c-statistic measures the overall model performance, and the ICC depicts the variance explained by the random intercept for the hospital.

Abbreviations: CI, confidence interval; ICC, intraclass correlation coefficient; OR, odds ratio; PNB, peripheral nerve block; CMS, Centers for Medicare & Medicaid Services.

The sequential PARs visualization provided the cumulative proportion of PNBs which could be explained by the regression model.^9^ All variables combined explained a considerably higher percentage of PNBs in THA (87%) compared to in TKA patients (63%). Starting with demographics and ending with hospital variables, in THA, the largest contributing factors were clinical (46%), followed by hospital (31%), demographic (7%), and socioeconomic variables (3%). In TKA, the largest contributors were also clinical (34%), followed by hospital (22%), demographic (3%) and socioeconomic variables (3%). If the order of the groups of variables was reversed (starting with the hospital), hospital variables explained a larger proportion in THA, but not in TKA (THA: 64%, TKA: 25%). The effect of clinical variables reduced in THA (19%) which illustrates the statistical interrelatedness of hospital and clinical variables.

### Association of PNBs with Secondary Outcomes

In THA, use of PNB did not significantly relate to CMS-defined complications (OR, 0.92 [95% CI, 0.78–1.10]), 90-day all-cause readmission (OR, 0.98 [95% CI, 0.87–1.10]) nor a length of stay >3 days (OR, 0.99 [95% CI, 0.89–1.11]; Table 4). In TKA, use of PNBs was significantly associated with a reduction in CMS-defined complications (OR, 0.82 [95% CI, 0.75–0.90]) and length of stay >3 days (OR, 0.90 [95% CI, 0.86–0.95]), however, no benefit was found on 90-day all-cause readmissions (OR, 0.98 [95% CI, 0.93–1.03]). The full models with adjustment factors can be found in Supplemental Digital Content 4, Supplemental Tables 3–8, http://links.lww.com/AA/E914.

### Model Performance

The mixed-effects regression models on PNB utilization produced high c-statistics (THA: 0.94, TKA: 0.86) and ICCs (THA: 0.67, TKA: 0.49; Table 2). The models for the secondary outcomes had lower c-statistics, varying from 0.66 to 0.82 in both THA and TKA (Table 4). ICCs were also lower, ranging from 0.02 to 0.16.

### Sensitivity Analysis

The addition of state-county ID as a random effect had no improvement on model fit, and estimates of SDI did not change (Supplemental Digital Content 4, Supplemental Tables 9–10, http://links.lww.com/AA/E914). In other words, we did not find evidence of variation in PNB use by county of residence beyond the SDI measure used. The inclusion of dual eligibility had no effect on PNB utilization (Supplemental Digital Content 4, Supplemental Table 11, http://links.lww.com/AA/E914), nor did multivariable estimates of outcomes change substantially (Supplemental Digital Content 4, Supplemental Tables 3–8, http://links.lww.com/AA/E914).

## DISCUSSION

### Main Findings

To the best of our knowledge, this is the first study to determine patterns of use and effectiveness of PNBs in THA and TKA patients using Medicare data. Contrary to our expectations, socioeconomic background (PAR: THA: 2%–3%, TKA: 3%) played a minor role in the observed variation in PNB utilization. Most variation was explained by clinical (THA: 19%–46%, TKA: 32%–34%) and hospital variables (THA: 31%–64%, TKA: 22%–25%). The PAR for clinical variables was driven by the decreased use of PNBs in patients with a nonosteoarthritis diagnosis, and in THA also by decreased use in the outpatient setting and patients with prior hospitalizations. In all, statistical relations in TKA echo those in THA, but the relative role of hospital-related effects is larger in THA. These findings illustrate that the strongest driving force behind disparities in the utilization of PNBs is based on practice differences (provider based) in semi- and nonelective arthroplasty patients.

Our study adds to the extensive evidence base^1,2^ that the use of PNB is associated with improved clinical outcome: in TKA patients, we found fewer complications and length of stay; differences in THA patients did not reach statistical significance.

### Comparison with Other Literature

Previous studies examined the impact of patient and hospital variables on the utilization of PNBs through standard regression techniques, which provide insufficient insight into the strength of the association. The PAR method used in this paper had additional value in this regard. Overall, our study found no clear evidence of disparities according to socioeconomic (SDI, race and ethnicity, and dual eligibility) variables. This finding diverges from a study by Keneally et al,^6^ which used ZIP-code-linked median income as SES indicator and found a higher income to significantly relate to increased utilization of PNBs in TKA. A reason for this discrepancy may be because our study applied a different comprehensive type of neighborhood SES indicator and at a different level of linkage. However, as we did not observe variation in PNB utilization according to the county of residence, it is unlikely variation by neighborhood indicators will be found in the current dataset. In TKA a weak association suggested Black patients (compared to White) received fewer PNBs. We do not believe this is strong evidence of an association, as the PAR analysis did not show substantial variation by race/ethnicity and this may also be the result of a type I error. In comparison, a recent study by Zhong et al^8^ used a private insurance database and found nonwhite compared to White TKA patients receiving PNBs less often. The contrasting findings highlight that the effect of socioeconomic variables also may differ by the studied population and type of health coverage, that is, private versus public.

PNB utilization was less in patients receiving THA and TKA for nonosteoarthritis indications. Fracture patients typically present in a nonelective setting which could limit the timely administration of PNBs. However, nonosteoarthritis indications for THA/TKA will also include a variety of (semi-)elective diagnoses such as posttraumatic osteoarthritis, osteonecrosis, and rheumatoid arthritis.^32,33^ Especially in TKA in which the number of fracture patients is relatively small, there is a large group of (semi-)elective patients in whom the abovementioned explanation may not suffice. Additionally, in THA patients with prior hospitalizations PNBs were used less often, which highlights another potential explanation for differential use: comorbid and/or semielective patients may fall outside of protocolized care pathways with as a consequence less use of PNBs.

Regional practice variations explained a large part of the variation in PNB use. In both THA and TKA patients, the Midwest region and urban hospitals are associated with increased utilization of PNBs. Strengthening the assertion that PNB utilization is largely determined by practice variations was that the addition of a random intercept for the hospital (which covers unspecified hospital effects) drastically improved model fit. Practice variations may indirectly lead to variations in use by socioeconomic or clinical variables, or vice versa. For example, different PNB utilization profiles may drive socioeconomic disparities in the background, because certain regions are inhabited by relatively less affluent and/or more Black patients, such as the South.^34,35^ This effect is probably limited, as we did not observe the interrelatedness of socioeconomic and hospital-related variables in the PAR analysis. Clinical and hospital-related variables, however, were statistically interrelated in THA patients, as the role of clinical variables reduced markedly after first accounting for hospital variables. In other words, particular patients (ie, comorbid/nonosteoarthritis) treated in hospitals/regions as reflected by the hospital-related variables received PNBs less often. We currently cannot determine the directionality of this effect. We think that survey data with targeted questions on barriers for use of PNBs per hospital/specialist group could provide valuable insights.^36^ This may also reveal if the overall socioeconomic or clinical profile of patients presenting at hospitals in certain regions affects PNB utilization at the policy level.

We expect the practitioners’ choice (surgeon or anesthesiologist) plays a key role in explaining these regional variations, which in turn largely depends on the training received and the experienced comfort with PNB utilization.^4^ For example, one study found that PNBs were applied more often in TKA patients when a board-certified anesthesiologist was present.^35^ In a study on the utilization of regional anesthesia for acute pain management among military anesthesiology residents and specialists, a potential barrier to apply PNBs was the lack of opportunities to practice during traing.^37^ The practice environment may differ largely by regions, and specific hospitals (urban, teaching) may have increased opportunities to practice PNB utilization for residents.

Diverging patterns between THA and TKA patients were observed with regard to hospital ownership: THA patients undergoing surgery in physician/proprietary (for-profit) hospitals had higher odds of receiving a PNB compared to voluntary or government (nonprofit) hospitals, while this was not the case in TKA patients. For-profit hospitals have different incentives and resources available compared to nonprofit hospitals. which may result in increased (earlier) adaptation of novel treatments with a slimmer evidence base.^38^ Supporting this notion is the fact that overall uptake of PNBs is far less in THA compared to TKA (8% vs 57%, respectively).

### Strengths and Limitations

This study has some limitations. Due to the observational nature of this study, we can only assess associations and not causal relations. Moreover, it is possible that potential confounders in the studied association were missed; in this scenario, currently observed associations may be overestimated. At the hospital level, separating ambulatory surgical centers owned by or affiliated with teaching hospitals might have resulted in more detailed insights into the effect of the resident-to-bed ratio. Secondly, our findings are only generalizable to the Medicare population; different patterns may exist for commercially insured patients, a growing group of arthroplasty recipients.^4,8^ Thirdly, the area-based social deprivation indicator may not entirely reflect deprivation at the individual level. Finally, PAR estimates represent the maximum attainable reduction in variation of PNB utilization; it is unlikely a change in clinical practice will eliminate all variation.

## CONCLUSIONS

In THA and TKA patients on Medicare, large variations exist in the utilization of PNBs by clinical (eg, indication for arthroplasty) and hospital variables, while demographic and socioeconomic variables played a limited role. These findings emphasize the substantial individual and hospital practice variation in PNB utilization. In light of the potential benefit of PNBs observed in our study and various other studies, we believe stakeholders should strive for more standardized provision of PNBs.

## ACKNOWLEDGMENTS

We would like to acknowledge Dr Brocha Stern for her help with the additional analyses performed during the review phase. Moreover, we would like to acknowledge the reviewers for their valuable and in-depth review of this work.

## DISCLOSURES

**Conflicts of Interest:** None. **Funding:** This work was funded by a travel grant provided by the EuroQol Research Foundation (1483-TVG) and the Erasmus Trustfonds (97030.2022.101.935/292/RB). The funders had no role in the design and conduct of the study; collection, management, analysis, and interpretation of the data; preparation, review, or approval of the manuscript; and decision to submit the manuscript for publication. **This manuscript was handled by:** Olubukola O. Nafiu, MD, FRCA, MS.

## Supplementary Material


