# Supplementary material for: Socioeconomic, Patient, and Hospital Determinants for the Utilization of Peripheral Nerve Blocks in Total Joint Arthroplasty
Source: Anesth Analg. 2025 Feb 14;140(3):675–86. doi: 10.1213/ANE.0000000000007107 (PMC11805468; doi:10.1213/ANE.0000000000007107)
Supplement: Supplementary file 1 [file ane-140-675-s001.docx]

**Supplemental Table 1: Procedural and diagnostic codes used to identify Peripheral Nerve Blocks and diagnosis for arthroplasty**

CPT, Diagnosis (ICD-9/10-CM), and procedural codes (ICD-9/10-PCS) used

Abbreviations: ICD = International Statistical Classification of Diseases and Related Health Problems, CM = Clinical Modification, PCS = Procedure Codes, CPT = Current Procedural Code

| Peripheral Nerve Blocks | CPT: 64445, 64446, 64447, 64448, 64449, 64450, 64520, 01991, 01992 ICD-9-PCS: 04.81, 05.31 ICD-10-PCS : 3E0T3BZ |
| --- | --- |
| Diagnosis osteoarthritis | Hip ICD-10-CM: M160, M1610, M1611, M1612, M169 Hip ICD-9-CM: 71515 Knee ICD-10-CM: M170, M1710, M1711, M1712, M179  Knee ICD-9-CM: 71516 |
