# Supplementary material for: Socioeconomic, Patient, and Hospital Determinants for the Utilization of Peripheral Nerve Blocks in Total Joint Arthroplasty
Source: Anesth Analg. 2025 Feb 14;140(3):675–86. doi: 10.1213/ANE.0000000000007107 (PMC11805468; doi:10.1213/ANE.0000000000007107)

Supplemental Figures 1-4: missingness plots

Plots visualizing the missingness patterns for independent variables according to each outcome. The cumulative missingness per variable is depicted at the top after the variable name. The outcome was showed no missingness per the definition and each patient having experienced the outcome was highlighted in red in each plot. Each dark grey line indicates a certain percentage of missingness, described in the legend of each plot. For length-of-stay, only patients who had inpatient surgery were included. In red indicates the observations with the respective outcome. Dual eligibility was only available for patients who had surgery from 2017 and onwards.

Abbreviations: PNB = Peripheral Nerve Block, CMS = Centers for Medicaid and Medicare Services

<u>Supplemental Figure 1: missingness according to PNBs</u>

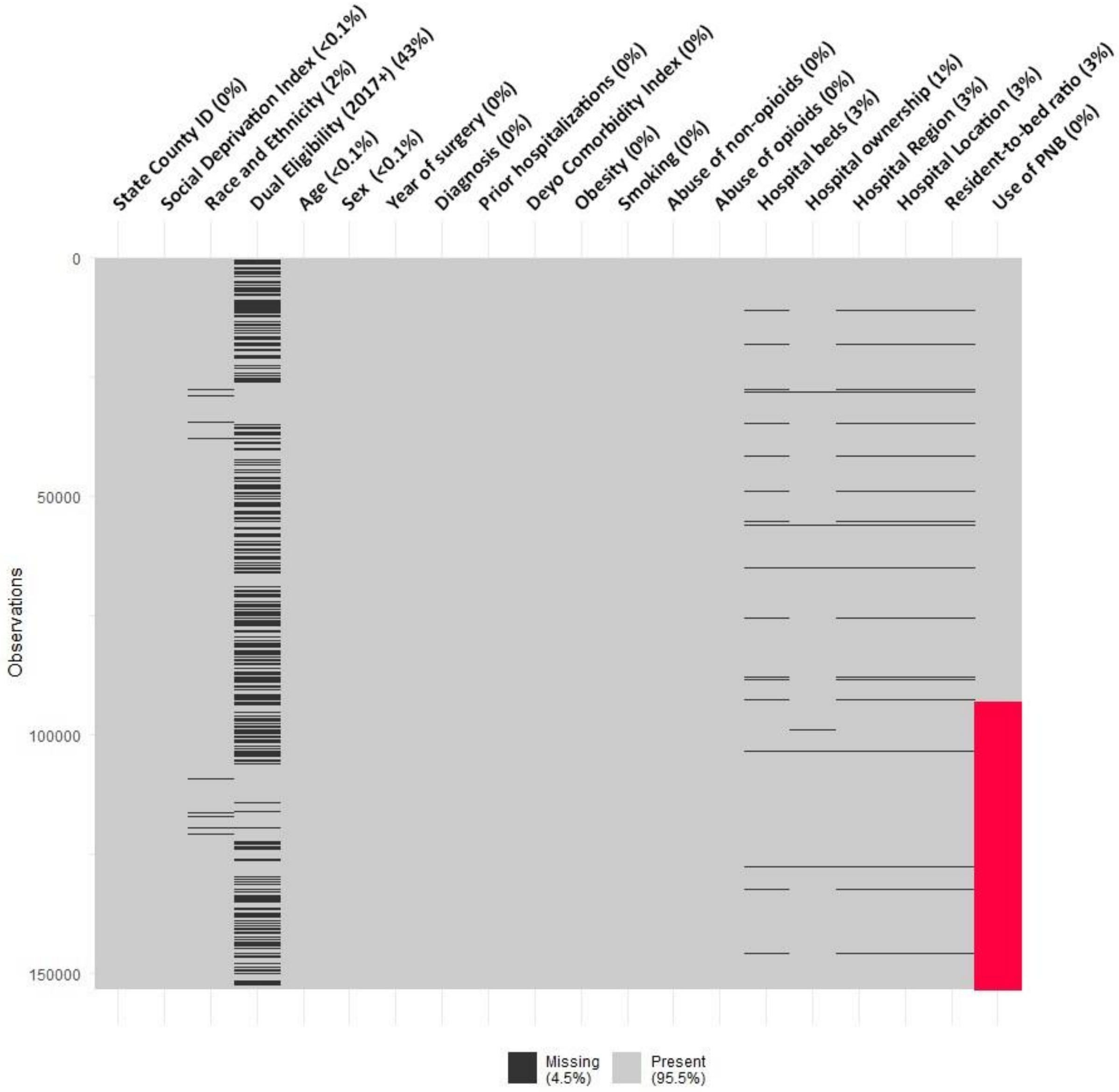

Supplemental Figure 2: missingness according to CMS-defined complications

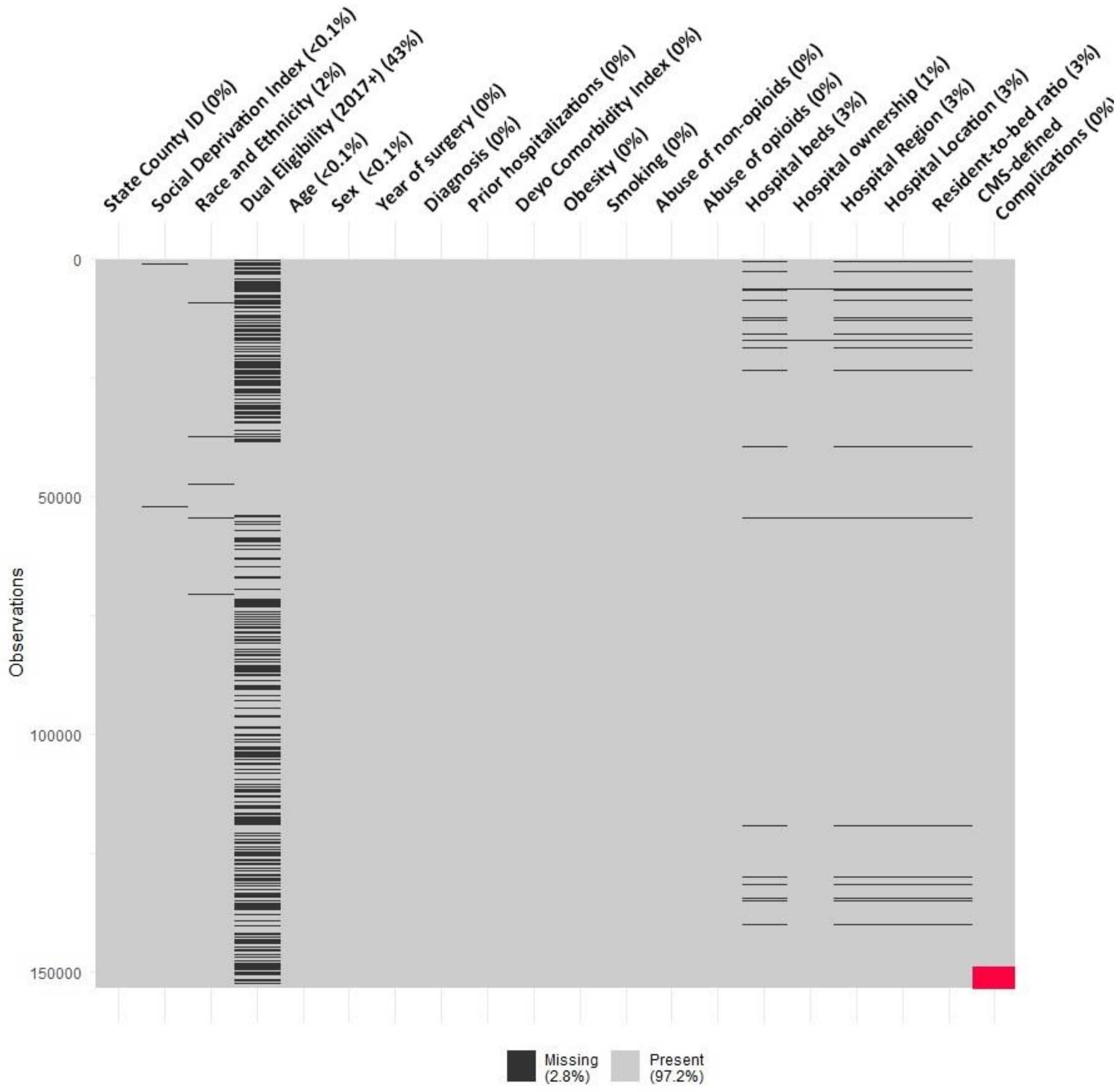

Supplemental Figure 3: missingness according to 90-day all-cause readmissions

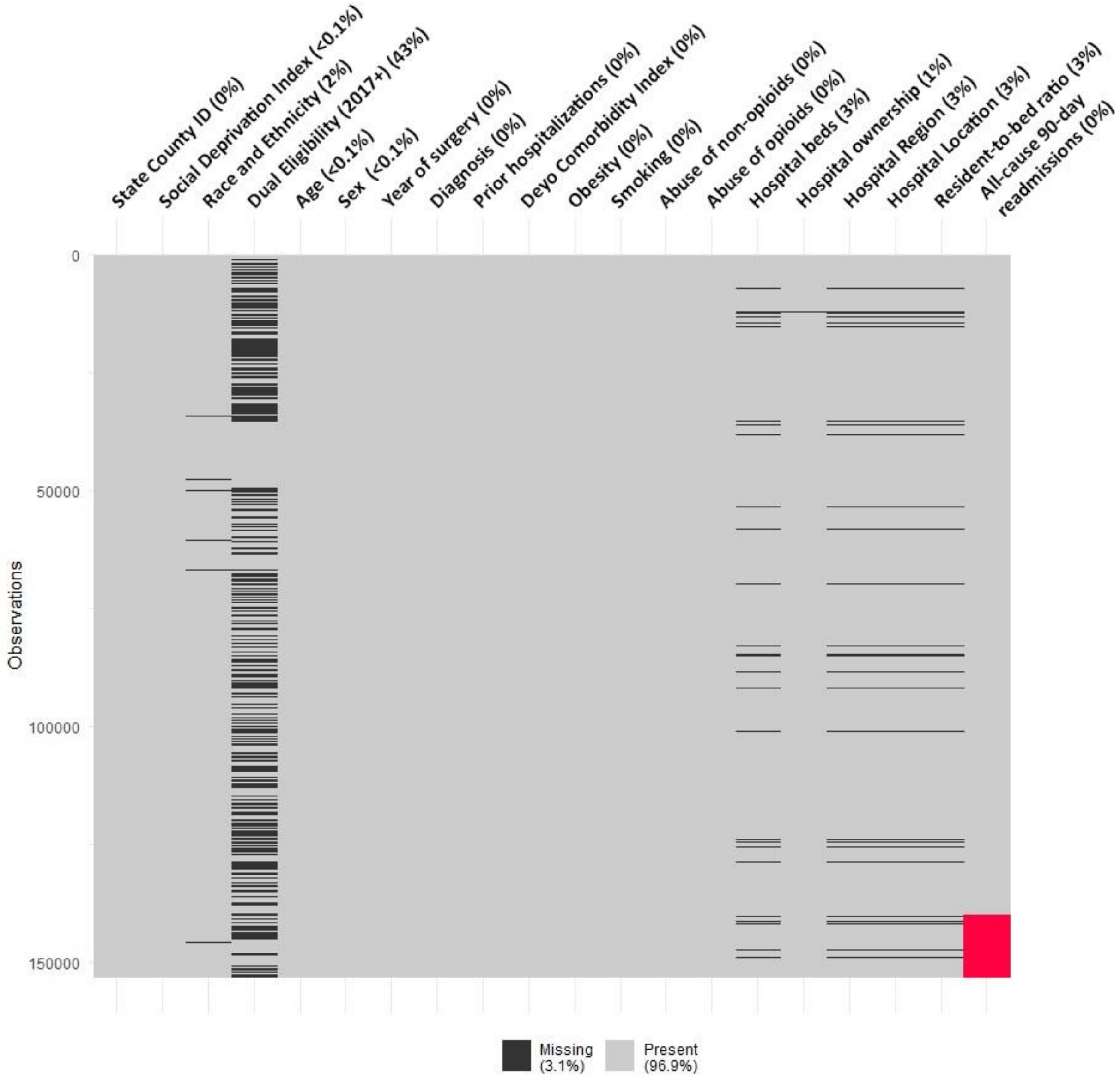

Supplemental Figure 4: missingness according to length of stay

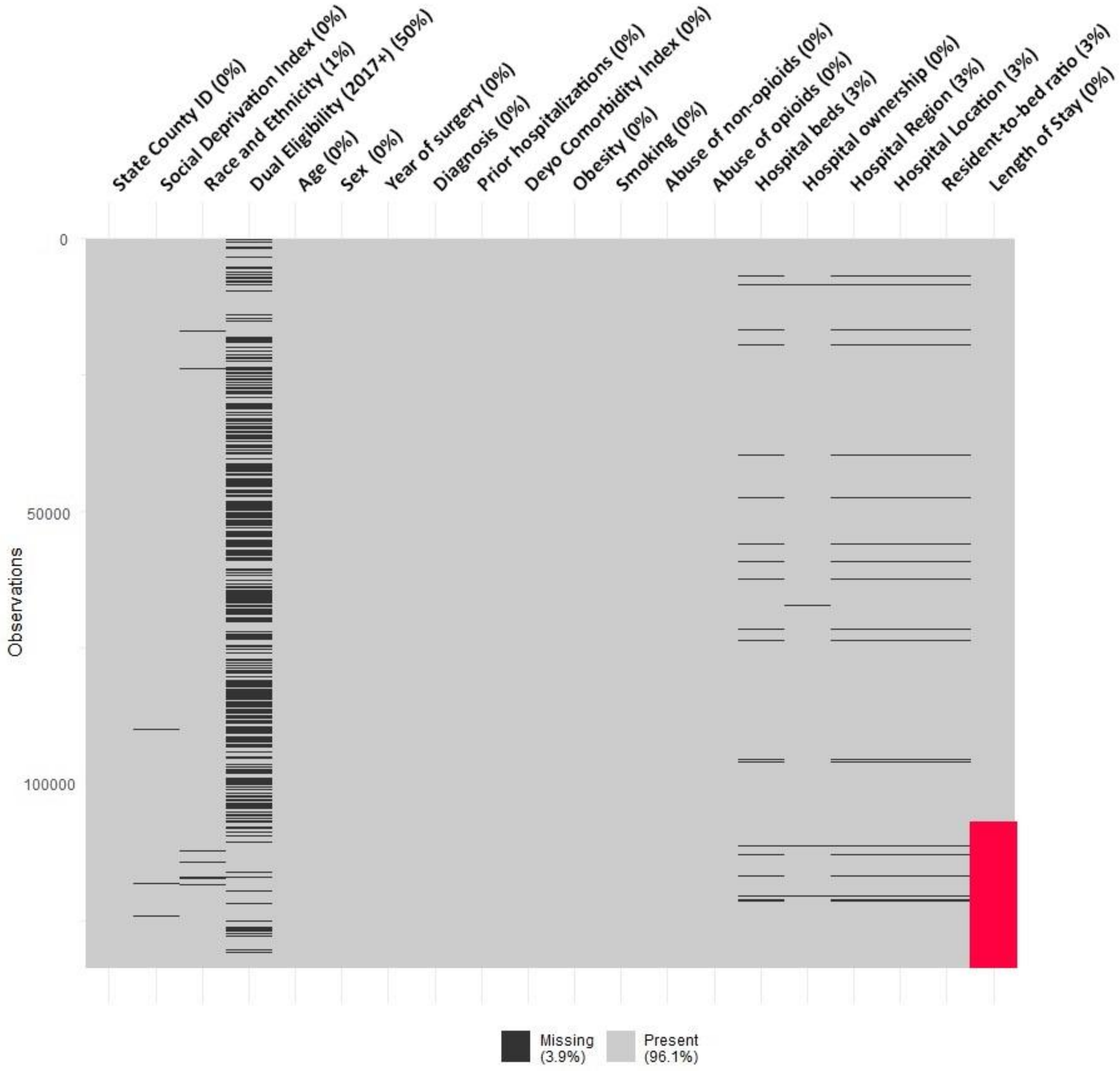

Supplement: Supplementary file 2 [file ane-140-675-s002.pdf]
