# Supplementary material for: Socioeconomic, Patient, and Hospital Determinants for the Utilization of Peripheral Nerve Blocks in Total Joint Arthroplasty
Source: Anesth Analg. 2025 Feb 14;140(3):675–86. doi: 10.1213/ANE.0000000000007107 (PMC11805468; doi:10.1213/ANE.0000000000007107)
Supplement: Supplementary file 3 [file ane-140-675-s003.docx]

**Supplemental Table 2: Procedural and diagnostic codes used to identify CMS-defined complications**

Diagnosis (ICD-9/10-CM) and procedural codes (ICD-9/10-PCS) used.

Abbreviations: CMS = Centers for Medicare and Medicaid Services, ICD = International Statistical Classification of Diseases and Related Health Problems, CM = Clinical Modification, PCS = Procedure Codes

| CMS-defined diagnosis* | ICD-9 and ICD-10 codes |
| --- | --- |
| Acute myocardial infarction (<7 days) | ICD-9-CM: 41001, 41010, 41011, 41021, 41031, 41040, 41041, 41042, 41051, 41061, 41071, 41081, 41091,  ICD-10-CM: I2101, I2102, I2109, I2111, I2119, I2121, I2129, I213, I214, I219 |
| Pneumonia | ICD-9-CM: 481, 485, 486, 4800, 4801, 4802, 4808, 4809, 4820, 4821, 4822, 48231, 48232, 48239, 48240, 48241, 48242, 48249, 48281, 48282, 48283, 48289, 4829, 48284, 4830, 4831, 4838, 4870, 4871, 48801, 48811, 48881, 5070, 99732, 99739,  ICD-10-CM: A481, J09X1, J1000, J1001, J1008, J1100, J1108, J120, J121, J122, J123, J1281, J1289, J129, J13, J14, J150, J151, J1520, J15211, J15212, J1529, J153, J154, J155, J156, J157, J158, J159, J160, J168, J180, J181, J188, J189, J690, J9589 |
| Sepsis/septicemia/shock | ICD-9-CM: 7907, 99590, 99591, 99592, 380, 3810, 3811, 3812, 3819, 382, 383, 3840, 3841, 3842, 3843, 3844, 3849, 388, 389, 99801, 99809, 31, 223, 271, 270, 9889, 1125, 78550, 78551, 78552, 99800,  ICD-10-CM: A021, A227, A267, A327, A400, A401, A403, A408, A409, A4101, A4102, A411, A412, A413, A414, A4150, A4151, A4152, A4153, A4159, A4181, A4189, A419, A427, A5486, B377, R570, R571, R578, R579, R6520, R6521, R7881, T8110XA, T8110XD, T8110XS, T8111XA, T8111XD, T8111XS, T8112XA, T8112XD, T8112XS, T8119XA, T8119XD, T8119XS, T8144XA, T8144XD, T8144XS |
| Surgical site bleeding | ICD-9-CM: 99811, 99812, 99813, 71910, 71915, 71916, 9093,  ICD-10-CM: D7802, D7822, E3602, G9732, G9752, I9742, I9752, I97620, J9562, J95831, K9162, K91841, L7602, L7622,  M2500, M25051, M25052, M25059, M25061, M25062, M25069, M96810, M96811, M96830, M96831, N9962, N99821, T8483XA, T8483XD, T8483XS, T888XXA, T888XXD, T888XXS)  and >=1 of    ICD-9-PCS: 3998, 8604,  ICD-10-PCS: 0H99X0Z, 0H99XZZ, 0H9HX0Z, 0H9HXZZ, 0H9JX0Z, 0H9JXZZ, 0H9KX0Z, 0H9KXZZ, 0H9LX0Z, 0H9LXZZ, 0J9900Z, 0J990ZZ, 0J9930Z, 0J993ZZ, 0J9B00Z, 0J9B0ZZ, 0J9B30Z, 0J9B3ZZ, 0J9C00Z, 0J9C0ZZ, 0J9C30Z, 0J9C3ZZ, 0J9L00Z, 0J9L0ZZ, 0J9L30Z, 0J9L3ZZ, 0J9M00Z, 0J9M0ZZ, 0J9M30Z, 0J9M3ZZ, 0J9N00Z, 0J9N0ZZ, 0J9N30Z, 0J9N3ZZ, 0J9P00Z, 0J9P0ZZ, 0J9P30Z, 0J9P3ZZ, 0W3J0ZZ, 0W3J3ZZ, 0W3J4ZZ, 0W3M0ZZ, 0W3M3ZZ, 0W3M4ZZ, 0W3N0ZZ, 0W3N3ZZ, 0W3N4ZZ, 0W3P0ZZ, 0W3P3ZZ, 0W3P4ZZ, 0W3P7ZZ, 0W3P8ZZ, 0W3R0ZZ, 0W3R3ZZ, 0W3R4ZZ, 0W3R7ZZ, 0W3R8ZZ, 0W9H00Z, 0W9H0ZZ, 0W9H30Z, 0W9H3ZZ, 0W9H40Z, 0W9H4ZZ, 0W9J70Z, 0W9J7ZX, 0W9J7ZZ, 0W9J80Z, 0W9J8ZX, 0W9J8ZZ, 0W9M00Z, 0W9M0ZZ, 0W9M30Z, 0W9M3ZZ, 0W9M40Z, 0W9M4ZZ, 0W9N00Z, 0W9N0ZZ, 0W9N30Z, 0W9N3ZZ, 0W9N40Z, 0W9N4ZZ, 0Y300ZZ, 0Y303ZZ, 0Y304ZZ, 0Y310ZZ, 0Y313ZZ, 0Y314ZZ, 0Y350ZZ, 0Y353ZZ, 0Y354ZZ, 0Y360ZZ, 0Y363ZZ, 0Y364ZZ, 0Y370ZZ, 0Y373ZZ, 0Y374ZZ, 0Y380ZZ, 0Y383ZZ, 0Y384ZZ, 0Y390ZZ, 0Y393ZZ, 0Y394ZZ, 0Y3B0ZZ, 0Y3B3ZZ, 0Y3B4ZZ, 0Y3C0ZZ, 0Y3C3ZZ, 0Y3C4ZZ, 0Y3D0ZZ, 0Y3D3ZZ, 0Y3D4ZZ, 0Y3F0ZZ, 0Y3F3ZZ, 0Y3F4ZZ, 0Y3G0ZZ, 0Y3G3ZZ, 0Y3G4ZZ, 0Y3H0ZZ, 0Y3H3ZZ, 0Y3H4ZZ, 0Y3J0ZZ, 0Y3J3ZZ, 0Y3J4ZZ, 0Y9000Z, 0Y900ZZ, 0Y9030Z, 0Y903ZZ, 0Y9040Z, 0Y904ZZ, 0Y9100Z, 0Y910ZZ, 0Y9130Z, 0Y913ZZ, 0Y9140Z, 0Y914ZZ, 0Y9500Z, 0Y950ZZ, 0Y9530Z, 0Y953ZZ, 0Y9540Z, 0Y954ZZ, 0Y9600Z, 0Y960ZZ, 0Y9630Z, 0Y963ZZ, 0Y9640Z, 0Y964ZZ, 0Y9700Z, 0Y970ZZ, 0Y9730Z, 0Y973ZZ, 0Y9740Z, 0Y974ZZ, 0Y9800Z, 0Y980ZZ, 0Y9830Z, 0Y983ZZ, 0Y9840Z, 0Y984ZZ, 0Y9900Z, 0Y990ZZ, 0Y9930Z, 0Y993ZZ, 0Y9940Z, 0Y994ZZ, 0Y9B00Z, 0Y9B0ZZ, 0Y9B30Z, 0Y9B3ZZ, 0Y9B40Z, 0Y9B4ZZ, 0Y9C00Z, 0Y9C0ZZ, 0Y9C30Z, 0Y9C3ZZ, 0Y9C40Z, 0Y9C4ZZ, 0Y9D00Z, 0Y9D0ZZ, 0Y9D30Z, 0Y9D3ZZ, 0Y9D40Z, 0Y9D4ZZ, 0Y9F00Z, 0Y9F0ZZ, 0Y9F30Z, 0Y9F3ZZ, 0Y9F40Z, 0Y9F4ZZ, 0Y9G00Z, 0Y9G0ZZ, 0Y9G30Z, 0Y9G3ZZ, 0Y9G40Z, 0Y9G4ZZ, 0Y9H00Z, 0Y9H0ZZ, 0Y9H30Z, 0Y9H3ZZ, 0Y9H40Z, 0Y9H4ZZ, 0Y9J00Z, 0Y9J0ZZ, 0Y9J30Z, 0Y9J3ZZ, 0Y9J40Z, 0Y9J4ZZ |
| Pulmonary embolism | ICD-9-CM: 4150, 41511, 41512, 41513, 41519,  ICD-10-CM: I2601, I2602, I2609, I2690, I2692, I2693, I2694, I2699, T81718A, T81718D, T81718S, T81719A, T81719D, T81719S, T8172XA, T8172XD, T8172XS, T82817A, T82817D, T82817S, T82818A, T82818D, T82818S, T8481XA, T8481XD, T8481XS |
| Death |  |
| Mechanical complications | ICD-9-CM: 99649, 99640, 99641, 99642, 99643, 99643, 99644, 99645, 99646, 99647, 99659,  ICD-10-CM: M9665, M96661, M96662, M96669, M96671, M96672, M96679, M9669, M9701XA, M9701XD, M9701XS, M9702XA, M9702XD, M9702XS, M9711XA, M9711XD, M9711XS, M9712XA, M9712XD, M9712XS, M978XXA, M978XXD, M978XXS, M979XXA, M979XXD, M979XXS, T84010A, T84010D, T84010S, T84011A, T84011D, T84011S, T84012A, T84012D, T84012S, T84013A, T84013D, T84013S, T84018A, T84018D, T84018S, T84019A, T84019D, T84019S, T84020A, T84020D, T84020S, T84021A, T84021D, T84021S, T84022A, T84022D, T84022S, T84023A, T84023D, T84023S, T84028A, T84028D, T84028S, T84029A, T84029D, T84029S, T84030A, T84030D, T84030S, T84031A, T84031D, T84031S, T84032A, T84032D, T84032S, T84033A, T84033D, T84033S, T84038A, T84038D, T84038S, T84039A, T84039D, T84039S, T84050A, T84050D, T84050S, T84051A, T84051D, T84051S, T84052A, T84052D, T84052S, T84053A, T84053D, T84053S, T84058A, T84058D, T84058S, T84059A, T84059D, T84059S, T84060A, T84060D, T84060S, T84061A, T84061D, T84061S, T84062A, T84062D, T84062S, T84063A, T84063D, T84063S, T84068A, T84068D, T84068S, T84069A, T84069D, T84069S, T84090A, T84090D, T84090S, T84091A, T84091D, T84091S, T84092A, T84092D, T84092S, T84093A, T84093D, T84093S, T84098A, T84098D, T84098S, T84099A, T84099D, T84099S, T84114A, T84114D, T84114S, T84115A, T84115D, T84115S, T84116A, T84116D, T84116S, T84117A, T84117D, T84117S, T84119A, T84119D, T84119S, T84124A, T84124D, T84124S, T84125A, T84125D, T84125S, T84126A, T84126D, T84126S, T84127A, T84127D, T84127S, T84129A, T84129D, T84129S, T84194A, T84194D, T84194S, T84195A, T84195D, T84195S, T84196A, T84196D, T84196S, T84197A, T84197D, T84197S, T84199A, T84199D, T84199S, T84318A, T84318D, T84318S, T84328A, T84328D, T84328S, T84398A, T84398D, T84398S, T84418A, T84418D, T84418S, T84428A, T84428D, T84428S, T84498A, T84498D, T84498S, T8489XA, T8489XD, T8489XS |
| Periprosthetic joint infection/wound infection | ICD-9-CM: 56738, 99830, 99831, 99832, 99833, 99859, 9986, 99666, 99667, 99677, 99678, 9989,  ICD-10-CM: K6811, K6819, T8130XA, T8130XD, T8130XS, T8131XA, T8131XD, T8131XS, T8132XA, T8132XD, T8132XS, T8133XA, T8133XD, T8133XS, T8140XA, T8140XD, T8140XS, T8141XA, T8141XD, T8141XS,  T8142XA, T8142XD, T8142XS, T8143XA, T8143XD, T8143XS, T814XXA, T814XXD, T814XXS, T8183XA, T8183XD, T8183XS, T8189XA, T8189XD, T8189XS, T819XXA, T819XXD, T819XXS, T8450XA, T8450XD, T8450XS, T8451XA, T8451XD, T8451XS, T8452XA, T8452XD, T8452XS, T8453XA, T8453XD, T8453XS,  T8454XA, T8454XD, T8454XS, T8459XA, T8459XD, T8459XS, T8460XA, T8460XD, T8460XS, T84620A, T84620D, T84620S, T84621A, T84621D, T84621S, T84622A, T84622D, T84622S, T84623A, T84623D, T84623S, T84624A, T84624D, T84624S, T84625A, T84625D, T84625S, T84629A, T84629D, T84629S, T847XXA  and >=1 of  ICD-9-PCS: 0070, 0071, 0072, 0073, 0080, 0081, 0082, 0083, 0084 , 7865 , 7866 , 7867 , 8005 , 8006 , 8009 , 8153, 8155 , 8159 , 8604 , 8622, 8628,  ICD-10-PCS: D7802, D7822, E3602, G9732, G9752, I9742, I9752, I97620, J9562, J95831, K9162, K91841, L7602, L7622, M2500, M25051, M25052, M25059, M25061, M25062, M25069, M96810, M96811, M96830, M96831, N9962, N99821, T8483XA, T8483XD, T8483XS, T888XXA, T888XXD, T888XXS, 0H99X0Z, 0H99XZZ, 0H9HX0Z, 0H9HXZZ, 0H9JX0Z, 0H9JXZZ, 0H9KX0Z, 0H9KXZZ, 0H9LX0Z, 0H9LXZZ, 0HB8XZZ, 0HB9XZZ, 0HBAXZZ, 0HBHXZZ, 0HBJXZZ, 0HBKXZZ, 0HBLXZZ, 0HD8XZZ, 0HD9XZZ, 0HDAXZZ, 0HDHXZZ, 0HDJXZZ, 0HDKXZZ, 0HDLXZZ, 0J9900Z, 0J990ZZ, 0J9930Z, 0J993ZZ, 0J9B00Z, 0J9B0ZZ, 0J9B30Z, 0J9B3ZZ, 0J9C00Z, 0J9C0ZZ, 0J9C30Z, 0J9C3ZZ, 0J9L00Z, 0J9L0ZZ, 0J9L30Z, 0J9L3ZZ, 0J9M00Z, 0J9M0ZZ, 0J9M30Z, 0J9M3ZZ, 0J9N00Z, 0J9N0ZZ, 0J9N30Z, 0J9N3ZZ, 0J9P00Z, 0J9P0ZZ, 0J9P30Z, 0J9P3ZZ, 0JB90ZZ, 0JB93ZZ, 0JBB0ZZ, 0JBB3ZZ, 0JBC0ZZ, 0JBC3ZZ, 0JBL0ZZ, 0JBL3ZZ, 0JBM0ZZ, 0JBM3ZZ, 0JBN0ZZ, 0JBN3ZZ, 0JBP0ZZ, 0JBP3ZZ, 0JD90ZZ, 0JD93ZZ, 0JDB0ZZ, 0JDB3ZZ, 0JDC0ZZ, 0JDC3ZZ, 0JDL0ZZ, 0JDL3ZZ, 0JDM0ZZ, 0JDM3ZZ, 0JDN0ZZ, 0JDN3ZZ, 0JDP0ZZ, 0JDP3ZZ, 0QP204Z, 0QP205Z, 0QP207Z, 0QP20JZ, 0QP20KZ, 0QP234Z, 0QP235Z, 0QP237Z, 0QP23JZ, 0QP23KZ, 0QP244Z, 0QP245Z, 0QP247Z, 0QP24JZ, 0QP24KZ, 0QP2X4Z, 0QP2X5Z, 0QP304Z, 0QP305Z, 0QP307Z, 0QP30JZ, 0QP30KZ, 0QP334Z, 0QP335Z, 0QP337Z, 0QP33JZ, 0QP33KZ, 0QP344Z, 0QP345Z, 0QP347Z, 0QP34JZ, 0QP34KZ, 0QP3X4Z, 0QP3X5Z, 0QP404Z, 0QP405Z, 0QP407Z, 0QP40JZ, 0QP40KZ, 0QP434Z, 0QP435Z, 0QP437Z, 0QP43JZ, 0QP43KZ, 0QP444Z, 0QP445Z, 0QP447Z, 0QP44JZ, 0QP44KZ, 0QP4X4Z, 0QP4X5Z, 0QP504Z, 0QP505Z, 0QP507Z, 0QP50JZ, 0QP50KZ, 0QP534Z, 0QP535Z, 0QP537Z, 0QP53JZ, 0QP53KZ, 0QP544Z, 0QP545Z, 0QP547Z, 0QP54JZ, 0QP54KZ, 0QP5X4Z, 0QP5X5Z, 0QP604Z, 0QP605Z, 0QP607Z, 0QP60JZ, 0QP60KZ, 0QP634Z, 0QP635Z, 0QP637Z, 0QP63JZ, 0QP63KZ, 0QP644Z, 0QP645Z, 0QP647Z, 0QP64JZ, 0QP64KZ, 0QP6X4Z, 0QP6X5Z, 0QP704Z, 0QP705Z, 0QP707Z, 0QP70JZ, 0QP70KZ, 0QP734Z, 0QP735Z, 0QP737Z, 0QP73JZ, 0QP73KZ, 0QP744Z, 0QP745Z, 0QP747Z, 0QP74JZ, 0QP74KZ, 0QP7X4Z, 0QP7X5Z, 0QP804Z, 0QP805Z, 0QP807Z, 0QP80JZ, 0QP80KZ, 0QP834Z, 0QP835Z, 0QP837Z, 0QP83JZ, 0QP83KZ, 0QP844Z, 0QP845Z, 0QP847Z, 0QP84JZ, 0QP84KZ, 0QP8X4Z, 0QP8X5Z, 0QP904Z, 0QP905Z, 0QP907Z, 0QP90JZ, 0QP90KZ, 0QP934Z, 0QP935Z, 0QP937Z, 0QP93JZ, 0QP93KZ, 0QP944Z, 0QP945Z, 0QP947Z, 0QP94JZ, 0QP94KZ, 0QP9X4Z, 0QP9X5Z, 0QPB04Z, 0QPB05Z, 0QPB07Z, 0QPB0JZ, 0QPB0KZ, 0QPB34Z, 0QPB35Z, 0QPB37Z, 0QPB3JZ, 0QPB3KZ, 0QPB44Z, 0QPB45Z, 0QPB47Z, 0QPB4JZ, 0QPB4KZ, 0QPBX4Z, 0QPBX5Z, 0QPC04Z, 0QPC05Z, 0QPC07Z, 0QPC0JZ, 0QPC0KZ, 0QPC34Z, 0QPC35Z, 0QPC37Z, 0QPC3JZ, 0QPC3KZ, 0QPC44Z, 0QPC45Z, 0QPC47Z, 0QPC4JZ, 0QPC4KZ, 0QPCX4Z, 0QPCX5Z, 0QPD04Z, 0QPD05Z, 0QPD07Z, 0QPD0JZ, 0QPD0KZ, 0QPD34Z, 0QPD35Z, 0QPD37Z, 0QPD3JZ, 0QPD3KZ, 0QPD44Z, 0QPD45Z, 0QPD47Z, 0QPD4JZ, 0QPD4KZ, 0QPDX4Z, 0QPDX5Z, 0QPF04Z, 0QPF05Z, 0QPF07Z, 0QPF0JZ, 0QPF0KZ, 0QPF34Z, 0QPF35Z, 0QPF37Z, 0QPF3JZ, 0QPF3KZ, 0QPF44Z, 0QPF45Z, 0QPF47Z, 0QPF4JZ, 0QPF4KZ, 0QPFX4Z, 0QPFX5Z, 0QPG04Z, 0QPG05Z, 0QPG07Z, 0QPG0JZ, 0QPG0KZ, 0QPG34Z, 0QPG35Z, 0QPG37Z, 0QPG3JZ, 0QPG3KZ, 0QPG44Z, 0QPG45Z, 0QPG47Z, 0QPG4JZ, 0QPG4KZ, 0QPGX4Z, 0QPGX5Z, 0QPH04Z, 0QPH05Z, 0QPH07Z, 0QPH0JZ, 0QPH0KZ, 0QPH34Z, 0QPH35Z, 0QPH37Z, 0QPH3JZ, 0QPH3KZ, 0QPH44Z, 0QPH45Z, 0QPH47Z, 0QPH4JZ, 0QPH4KZ, 0QPHX4Z, 0QPHX5Z, 0QPJ04Z, 0QPJ05Z, 0QPJ07Z, 0QPJ0JZ, 0QPJ0KZ, 0QPJ34Z, 0QPJ35Z, 0QPJ37Z, 0QPJ3JZ, 0QPJ3KZ, 0QPJ44Z, 0QPJ45Z, 0QPJ47Z, 0QPJ4JZ, 0QPJ4KZ, QPK04Z, 0QPK05Z, 0QPK07Z, 0QPK0JZ, 0QPK0KZ, 0QPK34Z, 0QPK35Z, 0QPK37Z, 0QPK3JZ, 0QPK3KZ, 0QPK44Z, 0QPK45Z, 0QPK47Z, 0QPK4JZ, 0QPK4KZ, 0QU207Z, 0QU20JZ, 0QU20KZ, 0QU237Z, 0QU23JZ, 0QU23KZ, 0QU247Z, 0QU24JZ, 0QU24KZ, 0QU307Z, QU30JZ, 0QU30KZ, 0QU337Z, 0QU33JZ, 0QU33KZ, 0QU347Z, 0QU34JZ, 0QU34KZ, 0QU607Z, 0QU60JZ, 0QU60KZ, 0QU637Z, 0QU63JZ, 0QU63KZ, 0QU647Z, 0QU64JZ, 0QU64KZ, 0QU707Z, 0QU70JZ, 0QU70KZ, 0QU737Z, 0QU73JZ, 0QU73KZ, 0QU747Z, 0QU74JZ, 0QU74KZ, 0QU807Z, 0QU80JZ, 0QU80KZ, 0QU837Z, 0QU83JZ, 0QU83KZ, 0QU847Z, 0QU84JZ, 0QU84KZ, 0QU907Z, 0QU90JZ, 0QU90KZ, 0QU937Z, 0QU93JZ, 0QU93KZ, 0QU947Z, 0QU94JZ, 0QU94KZ, 0QUB07Z, 0QUB0JZ, 0QUB0KZ, 0QUB37Z, 0QUB3JZ, 0QUB3KZ, 0QUB47Z, 0QUB4JZ, 0QUB4KZ, 0QUC07Z, 0QUC0JZ, 0QUC0KZ, 0QUC37Z, 0QUC3JZ, 0QUC3KZ, 0QUC47Z, 0QUC4JZ, 0QUC4KZ, 0QUD07Z, 0QUD0JZ, 0QUD0KZ, 0QUD37Z, 0QUD3JZ, 0QUD3KZ, 0QUD47Z, 0QUD4JZ, 0QUD4KZ, 0QUF07Z, 0QUF0JZ, 0QUF0KZ, 0QUF37Z, 0QUF3JZ, 0QUF3KZ, 0QUF47Z, 0QUF4JZ, 0QUF4KZ, 0QUG07Z, 0QUG0JZ, 0QUG0KZ, 0QUG37Z,  0QUG3JZ, 0QUG3KZ, 0QUG47Z, 0QUG4JZ, 0QUG4KZ, 0QUH07Z, 0QUH0JZ, 0QUH0KZ, 0QUH37Z, 0QUH3JZ, 0QUH3KZ, 0QUH47Z, 0QUH4JZ, 0QUH4KZ, 0QUJ07Z, 0QUJ0JZ, 0QUJ0KZ, 0QUJ37Z, 0QUJ3JZ, 0QUJ3KZ, 0QUJ47Z, 0QUJ4JZ, 0QUJ4KZ, 0QUK07Z, 0QUK0JZ, 0QUK0KZ, 0QUK37Z, 0QUK3JZ, 0QUK3KZ, 0QUK47Z, 0QUK4JZ, 0QUK4KZ, 0SP900Z, 0SP903Z, 0SP904Z, 0SP905Z, 0SP907Z, 0SP908Z, 0SP909Z, 0SP90BZ, 0SP90JZ, 0SP90KZ, 0SP930Z, 0SP933Z, 0SP934Z, 0SP935Z, 0SP937Z, 0SP938Z, 0SP93JZ, 0SP93KZ, 0SP940Z, 0SP943Z, 0SP944Z, 0SP945Z, 0SP947Z, 0SP948Z, 0SP94JZ, 0SP94KZ, 0SP9X0Z, 0SP9X3Z, 0SP9X4Z, 0SP9X5Z, 0SPA0JZ, 0SPA3JZ, 0SPA4JZ, 0SPB00Z, 0SPB03Z, 0SPB04Z, 0SPB05Z, 0SPB07Z, 0SPB08Z, 0SPB09Z, 0SPB0BZ, 0SPB0JZ, 0SPB0KZ, 0SPB30Z, 0SPB33Z, 0SPB34Z, 0SPB35Z, 0SPB37Z, 0SPB38Z, 0SPB3JZ, 0SPB3KZ, 0SPB40Z, 0SPB43Z, 0SPB44Z, 0SPB45Z, 0SPB47Z, 0SPB48Z, 0SPB4JZ, 0SPB4KZ, 0SPBX0Z, 0SPBX3Z, 0SPBX4Z, 0SPBX5Z, 0SPC00Z, 0SPC03Z, 0SPC04Z, 0SPC05Z, 0SPC07Z, 0SPC08Z, 0SPC09Z, 0SPC0JC, 0SPC0JZ, 0SPC0KZ, 0SPC30Z, 0SPC33Z, 0SPC34Z, 0SPC35Z, 0SPC37Z, 0SPC38Z, 0SPC3JC, 0SPC3JZ, 0SPC3KZ, 0SPC40Z, 0SPC43Z, 0SPC44Z, 0SPC45Z, 0SPC47Z, 0SPC48Z, 0SPC4JC, 0SPC4JZ, 0SPC4KZ, 0SPCX0Z, 0SPCX3Z, 0SPCX4Z, 0SPCX5Z, 0SPD00Z, 0SPD03Z, 0SPD04Z, 0SPD05Z, 0SPD07Z, 0SPD08Z, 0SPD09Z, 0SPD0JC, 0SPD0JZ, 0SPD0KZ, 0SPD30Z, 0SPD33Z, 0SPD34Z, 0SPD35Z, 0SPD37Z, 0SPD38Z, 0SPD3JC, 0SPD3JZ, 0SPD3KZ, 0SPD40Z, 0SPD43Z, 0SPD44Z, 0SPD45Z, 0SPD47Z, 0SPD48Z, 0SPD4JC, 0SPD4JZ, 0SPD4KZ, 0SPDX0Z, 0SPDX3Z, 0SPDX4Z, 0SPDX5Z, 0SPE0JZ, 0SPE3JZ, 0SPE4JZ, 0SPR0JZ, 0SPR3JZ, 0SPR4JZ, 0SPS0JZ, 0SPS3JZ, 0SPS4JZ, 0SPT0JZ, 0SPT3JZ, 0SPT4JZ, 0SPU0JZ, 0SPU3JZ, 0SPU4JZ, 0SPV0JZ, 0SPV3JZ, 0SPV4JZ, 0SPW0JZ, 0SPW3JZ, 0SPW4JZ, 0SU907Z, 0SU909Z, 0SU90BZ, 0SU90JZ, 0SU90KZ, 0SU937Z, 0SU93JZ, 0SU93KZ, 0SU947Z, 0SU94JZ, 0SU94KZ, 0SUA09Z, 0SUA0BZ, 0SUB07Z, 0SUB09Z, 0SUB0BZ, 0SUB0JZ, 0SUB0KZ, 0SUB37Z, 0SUB3JZ, 0SUB3KZ, 0SUB47Z, 0SUB4JZ, 0SUB4KZ, 0SUC07Z, 0SUC09C, 0SUC09Z, 0SUC0JZ, 0SUC0KZ, 0SUC37Z, 0SUC3JZ, 0SUC3KZ, 0SUC47Z, 0SUC4JZ, 0SUC4KZ, 0SUD07Z, 0SUD09C, 0SUD09Z, 0SUD0JZ, 0SUD0KZ, 0SUD37Z, 0SUD3JZ, 0SUD3KZ, 0SUD47Z, 0SUD4JZ, 0SUD4KZ, 0SUE09Z, 0SUE0BZ, 0SUR09Z, 0SUR0BZ, 0SUS09Z, 0SUS0BZ, 0SUT09Z, 0SUU09Z, 0SUV09Z, 0SUW09Z, 0SW900Z, 0SW903Z, 0SW904Z, 0SW905Z, 0SW907Z, 0SW908Z, 0SW909Z, 0SW90BZ, 0SW90JZ, 0SW90KZ, 0SW930Z, 0SW933Z, 0SW934Z, 0SW935Z, 0SW937Z, 0SW938Z, 0SW93JZ, 0SW93KZ, 0SW940Z, 0SW943Z, 0SW944Z, 0SW945Z, 0SW947Z, 0SW948Z, 0SW94JZ, 0SW94KZ, 0SW9X0Z, 0SW9X3Z, 0SW9X4Z, 0SW9X5Z, 0SW9X7Z, 0SW9X8Z, 0SW9XJZ, 0SW9XKZ, 0SWA0JZ, 0SWA3JZ, 0SWA4JZ, 0SWAXJZ, 0SWB00Z, 0SWB03Z, 0SWB04Z, 0SWB05Z, 0SWB07Z, 0SWB08Z, 0SWB09Z, 0SWB0BZ,  0SWB0JZ, 0SWB0KZ, 0SWB30Z, 0SWB33Z, 0SWB34Z, 0SWB35Z, 0SWB37Z, 0SWB38Z, 0SWB3JZ, 0SWB3KZ, 0SWB40Z, 0SWB43Z, 0SWB44Z, 0SWB45Z, 0SWB47Z, 0SWB48Z, 0SWB4JZ, 0SWB4KZ, 0SWBX0Z, 0SWBX3Z, 0SWBX4Z, 0SWBX5Z, 0SWBX7Z, 0SWBX8Z, 0SWBXJZ, 0SWBXKZ, 0SWC00Z, 0SWC03Z, 0SWC04Z, 0SWC05Z, 0SWC07Z, 0SWC08Z, 0SWC09Z, 0SWC0JC, 0SWC0JZ, 0SWC0KZ, 0SWC30Z, 0SWC33Z, 0SWC34Z, 0SWC35Z, 0SWC37Z, 0SWC38Z, 0SWC3JC, 0SWC3JZ, 0SWC3KZ, 0SWC40Z, 0SWC43Z, 0SWC44Z, 0SWC45Z, 0SWC47Z, 0SWC48Z, 0SWC4JC, 0SWC4JZ, 0SWC4KZ, 0SWCX0Z, 0SWCX3Z, 0SWCX4Z, 0SWCX5Z, 0SWCX7Z, 0SWCX8Z, 0SWCXJC, 0SWCXJZ, 0SWCXKZ, 0SWD00Z, 0SWD03Z, 0SWD04Z, 0SWD05Z, 0SWD07Z, 0SWD08Z, 0SWD09Z, 0SWD0JC, 0SWD0JZ, 0SWD0KZ, 0SWD30Z, 0SWD33Z, 0SWD34Z, 0SWD35Z, 0SWD37Z, 0SWD38Z, 0SWD3JC, 0SWD3JZ, 0SWD3KZ, 0SWD40Z, 0SWD43Z, 0SWD44Z, 0SWD45Z, 0SWD47Z, 0SWD48Z, 0SWD4JC, 0SWD4JZ, 0SWD4KZ, 0SWDX0Z, 0SWDX3Z, 0SWDX4Z, 0SWDX5Z, 0SWDX7Z, 0SWDX8Z, 0SWDXJC, 0SWDXJZ, 0SWDXKZ, 0SWE0JZ, 0SWE3JZ, 0SWE4JZ, 0SWEXJZ, 0SWR0JZ, 0SWR3JZ, 0SWR4JZ, 0SWS0JZ, 0SWS3JZ, 0SWS4JZ, 0SWT0JZ, 0SWT3JZ, 0SWT4JZ, 0SWTXJZ, 0SWU0JZ, 0SWU3JZ, 0SWU4JZ, 0SWUXJZ, 0SWV0JZ, 0SWV3JZ, 0SWV4JZ, 0SWVXJZ, 0SWW0JZ, 0SWW3JZ, 0SWW4JZ, 0SWWXJZ, 0W9H00Z, 0W9H0ZZ, 0W9H30Z, 0W9H3ZZ, 0W9H40Z, 0W9H4ZZ, 0W9J70Z, 0W9J7ZX, 0W9J7ZZ, 0W9J80Z, 0W9J8ZX, 0W9J8ZZ, 0W9M00Z, 0W9M0ZZ, 0W9M30Z, 0W9M3ZZ, 0W9M40Z, 0W9M4ZZ, 0W9N00Z, 0W9N0ZZ, 0W9N30Z, 0W9N3ZZ, 0W9N40Z, 0W9N4ZZ, 0Y9000Z, 0Y900ZZ, 0Y9030Z, 0Y903ZZ, 0Y9040Z, 0Y904ZZ, 0Y9100Z, 0Y910ZZ, 0Y9130Z, 0Y913ZZ, 0Y9140Z, 0Y914ZZ, 0Y9500Z, 0Y950ZZ, 0Y9530Z, 0Y953ZZ, 0Y9540Z, 0Y954ZZ, 0Y9600Z, 0Y960ZZ, 0Y9630Z, 0Y963ZZ, 0Y9640Z, 0Y964ZZ, 0Y9700Z, 0Y970ZZ, 0Y9730Z, 0Y973ZZ, 0Y9740Z, 0Y974ZZ, 0Y9800Z, 0Y980ZZ, 0Y9830Z, 0Y983ZZ, 0Y9840Z, 0Y984ZZ, 0Y9900Z, 0Y990ZZ, 0Y9930Z, 0Y993ZZ, 0Y9940Z, 0Y994ZZ, 0Y9B00Z, 0Y9B0ZZ, 0Y9B30Z, 0Y9B3ZZ, 0Y9B40Z, 0Y9B4ZZ, 0Y9C00Z, 0Y9C0ZZ, 0Y9C30Z, 0Y9C3ZZ, 0Y9C40Z, 0Y9C4ZZ, 0Y9D00Z, 0Y9D0ZZ, 0Y9D30Z, 0Y9D3ZZ, 0Y9D40Z, 0Y9D4ZZ, 0Y9F00Z, 0Y9F0ZZ, 0Y9F30Z, 0Y9F3ZZ, 0Y9F40Z, 0Y9F4ZZ, 0Y9G00Z, 0Y9G0ZZ, 0Y9G30Z, 0Y9G3ZZ, 0Y9G40Z, 0Y9G4ZZ, 0Y9H00Z, 0Y9H0ZZ, 0Y9H30Z, 0Y9H3ZZ, 0Y9H40Z, 0Y9H4ZZ, 0Y9J00Z, 0Y9J0ZZ, 0Y9J30Z, 0Y9J3ZZ, 0Y9J40Z, 0Y9J4ZZ, 3E1038Z, 3E10X8Z |

*****See also: https://qualitynet.cms.gov/inpatient/measures/complication/methodology
