# Supplementary material for: Socioeconomic, Patient, and Hospital Determinants for the Utilization of Peripheral Nerve Blocks in Total Joint Arthroplasty
Source: Anesth Analg. 2025 Feb 14;140(3):675–86. doi: 10.1213/ANE.0000000000007107 (PMC11805468; doi:10.1213/ANE.0000000000007107)
Supplement: Supplementary file 4 [file ane-140-675-s004.docx]

**Supplemental Tables 3 through 11**

- Supplemental Table 3 to 8: Mixed-effects models of PNBs on outcomes (CMS complications, 90-day all-cause readmissions, length of stay >3 days) in THA and TKA patients

  Analyses were conducted in a) the primary cohort (THA: n=52,000, TKA: n=93,448), and in b) a secondary cohort of patients whom had surgery in 2017 or later (THA: n=30,136, TKA: n=51,379). The secondary cohort was used to define dual eligibility status, which has only been included in the Medicare dataset since 2017. All analyses were repeated in this cohort including this variable.
- Supplemental Table 9 and 10: Mixed-effects models of variables of interest on PNB use in THA and TKA patients

  These models apply the primary cohort as in the main manuscript. The first model is the main model as depicted in the manuscript (model 1). Subsequently, SDI was removed, and state-county ID was added as additional random intercept (3-level model) (model 2). Finally, both were entered simultaneously (model 3). Coefficients and model fit were compared between models to determine whether the patient’s state-county ID captured any additional variance beyond the SDI in PNB utilization.
- Supplemental Table 11: Mixed-effects models of variables of interest including dual eligibility on PNB use in THA and TKA patients

  These models evaluate the additional contribution of dual eligibility (Medicare-Medicaid) status on the use of PNBs. The above-described secondary cohort of patients who had surgery in 2017 or later was used.
- Overall:
  Abbreviations: OR = odds ratio, PNB = peripheral nerve block, SDI = Social Deprivation Index
  For each model, a random intercept was included for the hospital. Reference categories were selected based on the category with the highest number of PNBs observed in univariable comparison of independent variables according to PNB utilization. This is not always the same for THA and TKA; we opted to choose the same reference category in those instances for comprehensibility.

**Supplemental Table 3: Mixed-effects models of PNBs on CMS complications in THA patients**

|  | **Primary cohort** | | | **Secondary cohort  (including dual eligibility)** | | |
| --- | --- | --- | --- | --- | --- | --- |
| **Variables** | **OR (95% CI)** | **P-value OR** | **P-value variable** | **OR (95% CI)** | **P-value OR** | **P-value variable** |
| **Intercept** | 0.07 (0.04, 0.12) | <0.001 | <0.001 | 0.05 (0.02, 0.09) | <0.001 | <0.001 |
| **Peripheral nerve blocks** | 0.92 (0.78, 1.10) | 0.357 | 0.357 | 0.98 (0.79, 1.22) | 0.882 | 0.882 |
| **Age** |  |  | <0.001 |  |  | <0.001 |
| 65-69 | 0.71 (0.62, 0.82) | <0.001 |  | 0.74 (0.62, 0.89) | 0.001 |  |
| 70-74 | 0.81 (0.72, 0.92) | 0.001 |  | 0.85 (0.72, 0.99) | 0.042 |  |
| 75-79 | *ref* |  |  | *ref* |  |  |
| 80-84 | 1.11 (0.97, 1.27) | 0.118 |  | 1.18 (0.98, 1.41) | 0.077 |  |
| >84 | 1.20 (1.03, 1.39) | 0.018 |  | 1.12 (0.91, 1.37) | 0.287 |  |
| **Female** | 1.10 (1.00, 1.21) | 0.041 | 0.041 | 1.10 (0.97, 1.24) | 0.145 | 0.145 |
| **Inpatient (vs. outpatient)** | 2.58 (1.98, 3.37) | <0.001 | <0.001 | 2.41 (1.84, 3.16) | <0.001 | <0.001 |
| **Diagnosis non-osteoarthritis** | 2.06 (1.87, 2.28) | <0.001 | <0.001 | 2.49 (2.17, 2.85) | <0.001 | <0.001 |
| **Prior hospitalizations** |  |  | <0.001 |  |  | <0.001 |
| 0 | *ref* |  |  | *ref* |  |  |
| 1 | 1.28 (1.14, 1.45) | <0.001 |  | 1.45 (1.24, 1.70) | <0.001 |  |
| >=2 | 1.62 (1.38, 1.90) | <0.001 |  | 1.52 (1.22, 1.90) | <0.001 |  |
| **Deyo index** |  |  | <0.001 |  |  | <0.001 |
| 0 | 0.61 (0.54, 0.69) | <0.001 |  | 0.63 (0.54, 0.74) | <0.001 |  |
| 1 | 0.74 (0.66, 0.84) | <0.001 |  | 0.80 (0.68, 0.94) | 0.006 |  |
| 2 | 0.76 (0.66, 0.86) | <0.001 |  | 0.79 (0.66, 0.94) | 0.007 |  |
| >=3 | *ref* |  |  | *ref* |  |  |
| **No obesity** | 0.77 (0.70, 0.85) | <0.001 | <0.001 | 0.81 (0.71, 0.92) | 0.002 | 0.002 |
| **No abuse of non-opioids** | 0.76 (0.64, 0.90) | 0.001 | 0.001 | 0.76 (0.62, 0.93) | 0.009 | 0.009 |
| **No abuse of opioids** | 0.53 (0.39, 0.72) | <0.001 | <0.001 | 0.55 (0.39, 0.77) | 0.001 | 0.001 |
| **No smoking** | 0.95 (0.82, 1.10) | 0.494 | 0.494 | 1.29 (0.87, 1.92) | 0.205 | 0.205 |
| **Social Deprivation Index** |  |  | 0.408 |  |  | 0.238 |
| Q1 (most affluent) | *ref* |  |  | *ref* |  |  |
| Q2,3,4 | 1.07 (0.94, 1.21) | 0.293 |  | 1.08 (0.92, 1.27) | 0.342 |  |
| Q5 (least affluent) | 1.11 (0.95, 1.29) | 0.189 |  | 0.95 (0.77, 1.17) | 0.638 |  |
| **Race and Ethnicity** |  |  | 0.482 |  |  | 0.562 |
| White | *ref* |  |  | *ref* |  |  |
| Black | 0.91 (0.72, 1.14) | 0.423 |  | 0.90 (0.66, 1.23) | 0.524 |  |
| Asian, Hispanic, North American Native, other | 0.85 (0.60, 1.20) | 0.354 |  | 0.82 (0.53, 1.27) | 0.373 |  |
| **Year of surgery** |  |  | 0.001 |  |  | 0.390 |
| 2013 | 0.70 (0.55, 0.87) | 0.002 |  |  |  |  |
| 2014 | 0.60 (0.48, 0.76) | <0.001 |  |  |  |  |
| 2015 | 0.77 (0.62, 0.96) | 0.018 |  |  |  |  |
| 2016 | 0.84 (0.68, 1.04) | 0.109 |  |  |  |  |
| 2017 | 0.90 (0.73, 1.11) | 0.324 |  | 0.92 (0.74, 1.14) | 0.433 |  |
| 2018 | 0.83 (0.67, 1.03) | 0.084 |  | 0.85 (0.68, 1.05) | 0.130 |  |
| 2019 | 0.80 (0.65, 0.99) | 0.039 |  | 0.82 (0.67, 1.02) | 0.073 |  |
| 2020 | 0.90 (0.73, 1.11) | 0.338 |  | 0.91 (0.73, 1.12) | 0.367 |  |
| 2021 | *ref* |  |  | *ref* |  |  |
| **Hospital beds** |  |  | 0.745 |  |  | 0.934 |
| >=500 | *ref* |  |  | *ref* |  |  |
| 150-499 | 0.97 (0.87, 1.09) | 0.625 |  | 1.01 (0.87, 1.17) | 0.869 |  |
| 0-150 | 0.94 (0.79, 1.11) | 0.453 |  | 0.98 (0.78, 1.22) | 0.830 |  |
| **Hospital ownership** |  |  | 0.002 |  |  | 0.036 |
| Government | 1.18 (0.99, 1.42) | 0.067 |  | 1.07 (0.84, 1.36) | 0.576 |  |
| Physician/Proprietary | *ref* |  |  | *ref* |  |  |
| Voluntary | 0.91 (0.79, 1.04) | 0.179 |  | 0.85 (0.71, 1.02) | 0.082 |  |
| **Region** |  |  | 0.263 |  |  | 0.931 |
| Northeast | 1.00 (0.86, 1.17) | 0.951 |  | 0.98 (0.80, 1.20) | 0.841 |  |
| South | 1.03 (0.91, 1.18) | 0.628 |  | 1.00 (0.84, 1.18) | 0.960 |  |
| Midwest | *ref* |  |  | *ref* |  |  |
| West | 0.90 (0.77, 1.05) | 0.170 |  | 0.94 (0.77, 1.15) | 0.563 |  |
| **Rurality** |  |  | 0.552 |  |  | 0.499 |
| Large Urban | *ref* |  |  | *ref* |  |  |
| Small Urban | 0.99 (0.89, 1.10) | 0.876 |  | 0.99 (0.86, 1.14) | 0.875 |  |
| Rural | 1.09 (0.91, 1.31) | 0.330 |  | 1.14 (0.90, 1.45) | 0.288 |  |
| **Resident-to-bed ratio** |  |  | 0.735 |  |  | 0.566 |
| No teaching | 0.94 (0.81, 1.11) | 0.476 |  | 0.90 (0.73, 1.10) | 0.310 |  |
| Minor teaching | 0.98 (0.82, 1.16) | 0.781 |  | 0.90 (0.72, 1.12) | 0.345 |  |
| Major teaching | *ref* |  |  | *ref* |  |  |
| **Dual Eligibility (yes)** |  |  |  | 1.52 (1.22, 1.88) | <0.001 | <0.001 |
| **C-statistic** | 0.71 |  |  | 0.75 |  |  |
| **ICC** | 0.04 |  |  | 0.05 |  |  |

**Supplemental Table 4: Mixed-effects models of PNBs on CMS complications in TKA patients**

|  | **Primary cohort** | | | **Secondary cohort  (including dual eligibility)** | | |
| --- | --- | --- | --- | --- | --- | --- |
| **Variables** | **OR (95% CI)** | **P-value OR** | **P-value variable** | **OR (95% CI)** | **P-value OR** | **P-value variable** |
| **Intercept** | 0.05 (0.03, 0.09) | <0.001 | <0.001 | 0.03 (0.02, 0.07) | <0.001 | <0.001 |
| **Peripheral nerve blocks** | 0.82 (0.75, 0.90) | <0.001 | <0.001 | 0.85 (0.75, 0.97) | 0.016 | 0.016 |
| **Age** |  |  | <0.001 |  |  | <0.001 |
| 65-69 | 0.79 (0.69, 0.90) | <0.001 |  | 0.69 (0.57, 0.83) | <0.001 |  |
| 70-74 | 0.88 (0.79, 1.00) | 0.041 |  | 0.72 (0.61, 0.85) | <0.001 |  |
| 75-79 | *ref* |  |  | *ref* |  |  |
| 80-84 | 1.21 (1.05, 1.39) | 0.007 |  | 1.10 (0.90, 1.33) | 0.353 |  |
| >84 | 1.42 (1.19, 1.71) | <0.001 |  | 1.16 (0.90, 1.51) | 0.259 |  |
| **Female** | 0.96 (0.87, 1.05) | 0.327 | 0.327 | 0.96 (0.84, 1.09) | 0.507 | 0.507 |
| **Inpatient (vs. outpatient)** | 1.88 (1.54, 2.29) | <0.001 | <0.001 | 1.72 (1.41, 2.11) | <0.001 | <0.001 |
| **Diagnosis non-osteoarthritis** | 1.65 (1.47, 1.85) | <0.001 | <0.001 | 2.80 (2.33, 3.37) | <0.001 | <0.001 |
| **Prior hospitalizations** |  |  | <0.001 |  |  | <0.001 |
| 0 | *ref* |  |  | *ref* |  |  |
| 1 | 1.25 (1.10, 1.42) | 0.001 |  | 1.15 (0.95, 1.39) | 0.140 |  |
| >=2 | 1.92 (1.60, 2.30) | <0.001 |  | 1.88 (1.47, 2.39) | <0.001 |  |
| **Deyo index** |  |  | <0.001 |  |  | <0.001 |
| 0 | 0.54 (0.48, 0.61) | <0.001 |  | 0.54 (0.46, 0.64) | <0.001 |  |
| 1 | 0.72 (0.63, 0.81) | <0.001 |  | 0.70 (0.59, 0.83) | <0.001 |  |
| 2 | 0.85 (0.75, 0.97) | 0.013 |  | 0.80 (0.66, 0.96) | 0.016 |  |
| >=3 | *ref* |  |  | *ref* |  |  |
| **No obesity** | 0.78 (0.71, 0.85) | <0.001 | <0.001 | 0.79 (0.70, 0.91) | 0.001 | 0.001 |
| **No abuse of non-opioids** | 0.80 (0.66, 0.98) | 0.034 | 0.034 | 0.77 (0.59, 1.00) | 0.047 | 0.047 |
| **No abuse of opioids** | 0.63 (0.43, 0.92) | 0.016 | 0.016 | 0.67 (0.44, 1.02) | 0.059 | 0.059 |
| **No smoking** | 0.76 (0.66, 0.88) | <0.001 | <0.001 | 1.17 (0.69, 1.97) | 0.564 | 0.564 |
| **Social Deprivation Index** |  |  | 0.324 |  |  | 0.082 |
| Q1 (most affluent) | *ref* |  |  | *ref* |  |  |
| Q2,3,4 | 1.02 (0.90, 1.15) | 0.783 |  | 1.10 (0.93, 1.32) | 0.271 |  |
| Q5 (least affluent) | 1.10 (0.95, 1.28) | 0.198 |  | 1.26 (1.02, 1.56) | 0.030 |  |
| **Race and Ethnicity** |  |  | 0.070 |  |  | 0.003 |
| White | *ref* |  |  | *ref* |  |  |
| Black | 1.18 (0.98, 1.42) | 0.080 |  | 1.26 (0.98, 1.62) | 0.073 |  |
| Asian, Hispanic, North American Native, other | 0.83 (0.63, 1.08) | 0.159 |  | 0.53 (0.34, 0.82) | 0.005 |  |
| **Year of surgery** |  |  | 0.019 |  |  | 0.051 |
| 2013 | 1.05 (0.82, 1.35) | 0.687 |  |  |  |  |
| 2014 | 0.92 (0.72, 1.19) | 0.532 |  |  |  |  |
| 2015 | 0.92 (0.71, 1.18) | 0.493 |  |  |  |  |
| 2016 | 1.15 (0.90, 1.46) | 0.263 |  |  |  |  |
| 2017 | 1.27 (1.00, 1.61) | 0.051 |  | 1.33 (1.05, 1.70) | 0.019 |  |
| 2018 | 1.12 (0.88, 1.42) | 0.360 |  | 1.16 (0.91, 1.47) | 0.223 |  |
| 2019 | 1.01 (0.80, 1.29) | 0.919 |  | 1.05 (0.82, 1.33) | 0.713 |  |
| 2020 | 1.10 (0.86, 1.41) | 0.447 |  | 1.10 (0.85, 1.41) | 0.471 |  |
| 2021 | *ref* |  |  | *ref* |  |  |
| **Hospital beds** |  |  | 0.159 |  |  | 0.662 |
| >=500 | *ref* |  |  | *ref* |  |  |
| 150-499 | 0.93 (0.83, 1.04) | 0.202 |  | 0.93 (0.80, 1.08) | 0.364 |  |
| 0-150 | 0.86 (0.73, 1.01) | 0.062 |  | 0.96 (0.78, 1.18) | 0.678 |  |
| **Hospital ownership** |  |  | 0.908 |  |  | 0.406 |
| Government | 0.99 (0.82, 1.20) | 0.918 |  | 0.98 (0.77, 1.24) | 0.840 |  |
| Physician/Proprietary | *ref* |  |  | *ref* |  |  |
| Voluntary | 0.97 (0.85, 1.11) | 0.678 |  | 0.90 (0.76, 1.07) | 0.217 |  |
| **Region** |  |  | 0.868 |  |  | 0.957 |
| Northeast | 1.01 (0.86, 1.18) | 0.932 |  | 1.02 (0.82, 1.25) | 0.883 |  |
| South | 1.04 (0.91, 1.18) | 0.590 |  | 0.98 (0.83, 1.17) | 0.849 |  |
| Midwest | *ref* |  |  | *ref* |  |  |
| West | 1.06 (0.91, 1.24) | 0.424 |  | 1.03 (0.84, 1.27) | 0.753 |  |
| **Rurality** |  |  | 0.016 |  |  | 0.320 |
| Large Urban | *ref* |  |  | *ref* |  |  |
| Small Urban | 0.87 (0.78, 0.97) | 0.010 |  | 0.92 (0.80, 1.06) | 0.251 |  |
| Rural | 1.03 (0.86, 1.22) | 0.761 |  | 1.07 (0.85, 1.35) | 0.578 |  |
| **Resident-to-bed ratio** |  |  | 0.646 |  |  | 0.332 |
| No teaching | 1.00 (0.85, 1.19) | 0.974 |  | 1.07 (0.86, 1.34) | 0.542 |  |
| Minor teaching | 0.95 (0.79, 1.13) | 0.545 |  | 0.94 (0.74, 1.20) | 0.628 |  |
| Major teaching | *ref* |  |  | *ref* |  |  |
| **Dual Eligibility (yes)** |  |  |  | 1.43 (1.13, 1.79) | 0.002 | 0.002 |
| **C-statistic** | 0.70 |  |  | 0.70 |  |  |
| **ICC** | 0.04 |  |  | 0.01 |  |  |

**Supplemental Table 5: Mixed-effects models of PNBs on 90-day all-cause readmissions in THA patients**

|  | **Primary cohort** | | | **Secondary cohort  (including dual eligibility)** | | |
| --- | --- | --- | --- | --- | --- | --- |
| **Variables** | **OR (95% CI)** | **P-value OR** | **P-value variable** | **OR (95% CI)** | **P-value OR** | **P-value variable** |
| **Intercept** | 0.26 (0.18, 0.36) | <0.001 | <0.001 | 0.24 (0.16, 0.37) | <0.001 | <0.001 |
| **Peripheral nerve blocks** | 0.98 (0.87, 1.10) | 0.738 | 0.738 | 1.02 (0.87, 1.18) | 0.834 | 0.834 |
| **Age** |  |  | <0.001 |  |  | <0.001 |
| 65-69 | 0.75 (0.68, 0.82) | <0.001 |  | 0.76 (0.67, 0.87) | <0.001 |  |
| 70-74 | 0.86 (0.79, 0.93) | <0.001 |  | 0.90 (0.80, 1.01) | 0.075 |  |
| 75-79 | *ref* |  |  | *ref* |  |  |
| 80-84 | 1.22 (1.11, 1.34) | <0.001 |  | 1.26 (1.10, 1.43) | 0.001 |  |
| >84 | 1.43 (1.29, 1.58) | <0.001 |  | 1.40 (1.21, 1.61) | <0.001 |  |
| **Female** | 0.94 (0.89, 1.00) | 0.068 | 0.068 | 0.92 (0.85, 1.00) | 0.059 | 0.059 |
| **Inpatient (vs. outpatient)** | 1.41 (1.21, 1.65) | <0.001 | <0.001 | 1.35 (1.15, 1.59) | <0.001 | <0.001 |
| **Diagnosis non-osteoarthritis** | 1.44 (1.34, 1.55) | <0.001 | <0.001 | 1.74 (1.57, 1.93) | <0.001 | <0.001 |
| **Prior hospitalizations** |  |  | <0.001 |  |  | <0.001 |
| 0 | *ref* |  |  | *ref* |  |  |
| 1 | 1.39 (1.28, 1.51) | <0.001 |  | 1.50 (1.34, 1.68) | <0.001 |  |
| >=2 | 2.25 (2.01, 2.51) | <0.001 |  | 2.08 (1.78, 2.43) | <0.001 |  |
| **Deyo index** |  |  | <0.001 |  |  | <0.001 |
| 0 | 0.56 (0.51, 0.61) | <0.001 |  | 0.54 (0.49, 0.61) | <0.001 |  |
| 1 | 0.73 (0.67, 0.79) | <0.001 |  | 0.75 (0.67, 0.84) | <0.001 |  |
| 2 | 0.74 (0.67, 0.81) | <0.001 |  | 0.76 (0.67, 0.86) | <0.001 |  |
| >=3 | *ref* |  |  | *ref* |  |  |
| **No obesity** | 0.85 (0.79, 0.91) | <0.001 | <0.001 | 0.88 (0.80, 0.97) | 0.009 | 0.009 |
| **No abuse of non-opioids** | 0.77 (0.68, 0.87) | <0.001 | <0.001 | 0.81 (0.70, 0.95) | 0.008 | 0.008 |
| **No abuse of opioids** | 0.49 (0.39, 0.62) | <0.001 | <0.001 | 0.49 (0.38, 0.63) | <0.001 | <0.001 |
| **No smoking** | 1.03 (0.93, 1.14) | 0.586 | 0.586 | 1.07 (0.81, 1.42) | 0.619 | 0.619 |
| **Social Deprivation Index** |  |  | 0.085 |  |  | 0.520 |
| Q1 (most affluent) | *ref* |  |  | *ref* |  |  |
| Q2,3,4 | 1.07 (0.98, 1.16) | 0.123 |  | 1.07 (0.95, 1.19) | 0.265 |  |
| Q5 (least affluent) | 1.12 (1.01, 1.25) | 0.027 |  | 1.04 (0.90, 1.20) | 0.610 |  |
| **Race and Ethnicity** |  |  | 0.555 |  |  | 0.628 |
| White | *ref* |  |  | *ref* |  |  |
| Black | 1.01 (0.87, 1.17) | 0.931 |  | 1.02 (0.83, 1.25) | 0.857 |  |
| Asian, Hispanic, North American Native, other | 0.88 (0.69, 1.11) | 0.280 |  | 0.86 (0.63, 1.18) | 0.348 |  |
| **Year of surgery** |  |  | 0.018 |  |  | 0.621 |
| 2013 | 1.14 (0.97, 1.33) | 0.121 |  |  |  |  |
| 2014 | 1.04 (0.88, 1.22) | 0.646 |  |  |  |  |
| 2015 | 1.12 (0.96, 1.31) | 0.153 |  |  |  |  |
| 2016 | 1.11 (0.95, 1.29) | 0.192 |  |  |  |  |
| 2017 | 0.95 (0.81, 1.11) | 0.513 |  | 0.96 (0.82, 1.13) | 0.637 |  |
| 2018 | 1.02 (0.88, 1.19) | 0.787 |  | 1.04 (0.89, 1.21) | 0.612 |  |
| 2019 | 0.94 (0.81, 1.10) | 0.444 |  | 0.96 (0.82, 1.12) | 0.619 |  |
| 2020 | 0.95 (0.82, 1.10) | 0.510 |  | 0.96 (0.82, 1.11) | 0.542 |  |
| 2021 | *ref* |  |  | *ref* |  |  |
| **Hospital beds** |  |  | 0.400 |  |  | 0.339 |
| >=500 |  |  |  |  |  |  |
| 150-499 | 1.05 (0.97, 1.13) | 0.246 |  | 1.07 (0.97, 1.19) | 0.174 |  |
| 0-150 | 1.00 (0.89, 1.12) | 0.956 |  | 1.01 (0.87, 1.18) | 0.874 |  |
| **Hospital ownership** |  |  | 0.013 |  |  | 0.046 |
| Government | 0.96 (0.85, 1.09) | 0.562 |  | 0.88 (0.74, 1.04) | 0.127 |  |
| Physician/Proprietary | *ref* |  |  | *ref* |  |  |
| Voluntary | 0.88 (0.80, 0.96) | 0.006 |  | 0.86 (0.76, 0.97) | 0.013 |  |
| **Region** |  |  | 0.004 |  |  | 0.279 |
| Northeast | 1.07 (0.97, 1.19) | 0.177 |  | 0.98 (0.85, 1.12) | 0.753 |  |
| South | 1.08 (0.99, 1.18) | 0.080 |  | 1.01 (0.89, 1.13) | 0.912 |  |
| Midwest | *ref* |  |  | *ref* |  |  |
| West | 0.92 (0.83, 1.02) | 0.120 |  | 0.89 (0.78, 1.03) | 0.114 |  |
| **Rurality** |  |  | 0.013 |  |  | 0.250 |
| Large Urban | *ref* |  |  | *ref* |  |  |
| Small Urban | 0.90 (0.84, 0.97) | 0.004 |  | 0.93 (0.85, 1.02) | 0.136 |  |
| Rural | 0.97 (0.85, 1.10) | 0.590 |  | 1.03 (0.86, 1.22) | 0.772 |  |
| **Resident-to-bed ratio** |  |  | 0.773 |  |  | 0.456 |
| No teaching | 0.97 (0.88, 1.08) | 0.636 |  | 0.92 (0.79, 1.06) | 0.232 |  |
| Minor teaching | 1.00 (0.89, 1.12) | 0.975 |  | 0.96 (0.82, 1.11) | 0.556 |  |
| Major teaching | *ref* |  |  | *ref* |  |  |
| **Dual Eligibility (yes)** |  |  |  | 1.46 (1.24, 1.72) | <0.001 | <0.001 |
| **C-statistic** | 0.67 |  |  | 0.69 |  |  |
| **ICC** | 0.01 |  |  | 0.02 |  |  |

**Supplemental Table 6: Mixed-effects models of PNBs on 90-day all-cause readmissions in TKA patients**

|  | **Primary cohort** | | | **Secondary cohort  (including dual eligibility)** | | |
| --- | --- | --- | --- | --- | --- | --- |
| **Variables** | **OR (95% CI)** | **P-value OR** | **P-value variable** | **OR (95% CI)** | **P-value OR** | **P-value variable** |
| **Intercept** | 0.31 (0.22, 0.42) | <0.001 | <0.001 | 0.23 (0.15, 0.34) | <0.001 | <0.001 |
| **Peripheral nerve blocks** | 0.98 (0.93, 1.03) | 0.380 | 0.380 | 0.96 (0.89, 1.03) | 0.209 | 0.209 |
| **Age** |  |  | <0.001 |  |  | <0.001 |
| 65-69 | 0.74 (0.69, 0.79) | <0.001 |  | 0.71 (0.65, 0.79) | <0.001 |  |
| 70-74 | 0.84 (0.79, 0.90) | <0.001 |  | 0.85 (0.78, 0.93) | <0.001 |  |
| 75-79 | *ref* |  |  | *ref* |  |  |
| 80-84 | 1.15 (1.07, 1.25) | <0.001 |  | 1.17 (1.05, 1.30) | 0.006 |  |
| >84 | 1.62 (1.47, 1.78) | <0.001 |  | 1.64 (1.43, 1.88) | <0.001 |  |
| **Female** | 0.84 (0.80, 0.88) | <0.001 | <0.001 | 0.83 (0.77, 0.89) | <0.001 | <0.001 |
| **Inpatient (vs. outpatient)** | 1.32 (1.20, 1.45) | <0.001 | <0.001 | 1.29 (1.17, 1.42) | <0.001 | <0.001 |
| **Diagnosis non-osteoarthritis** | 1.16 (1.08, 1.24) | <0.001 | <0.001 | 1.52 (1.34, 1.72) | <0.001 | <0.001 |
| **Prior hospitalizations** |  |  | <0.001 |  |  | <0.001 |
| 0 | *ref* |  |  | *ref* |  |  |
| 1 | 1.39 (1.30, 1.49) | <0.001 |  | 1.26 (1.14, 1.40) | <0.001 |  |
| >=2 | 2.45 (2.21, 2.72) | <0.001 |  | 2.31 (2.00, 2.67) | <0.001 |  |
| **Deyo index** |  |  | <0.001 |  |  | <0.001 |
| 0 | 0.55 (0.52, 0.59) | <0.001 |  | 0.54 (0.50, 0.60) | <0.001 |  |
| 1 | 0.70 (0.66, 0.75) | <0.001 |  | 0.69 (0.63, 0.76) | <0.001 |  |
| 2 | 0.75 (0.70, 0.81) | <0.001 |  | 0.72 (0.65, 0.80) | <0.001 |  |
| >=3 | *ref* |  |  | *ref* |  |  |
| **No obesity** | 0.86 (0.82, 0.91) | <0.001 | <0.001 | 0.87 (0.81, 0.93) | <0.001 | <0.001 |
| **No abuse of non-opioids** | 0.82 (0.73, 0.92) | 0.001 | 0.001 | 0.78 (0.67, 0.90) | 0.001 | 0.001 |
| **No abuse of opioids** | 0.56 (0.45, 0.70) | <0.001 | <0.001 | 0.54 (0.43, 0.69) | <0.001 | <0.001 |
| **No smoking** | 0.84 (0.78, 0.91) | <0.001 | <0.001 | 1.21 (0.90, 1.62) | 0.213 | 0.213 |
| **Social Deprivation Index** |  |  | 0.351 |  |  | 0.364 |
| Q1 (most affluent) | *ref* |  |  | *ref* |  |  |
| Q2,3,4 | 1.00 (0.94, 1.08) | 0.891 |  | 1.05 (0.96, 1.15) | 0.280 |  |
| Q5 (least affluent) | 1.05 (0.97, 1.14) | 0.235 |  | 1.09 (0.97, 1.22) | 0.160 |  |
| **Race and Ethnicity** |  |  | 0.235 |  |  | 0.068 |
| White | *ref* |  |  | *ref* |  |  |
| Black | 1.06 (0.95, 1.18) | 0.267 |  | 1.08 (0.93, 1.26) | 0.300 |  |
| Asian, Hispanic, North American Native, other | 0.92 (0.80, 1.05) | 0.219 |  | 0.81 (0.67, 1.00) | 0.046 |  |
| **Year of surgery** |  |  | <0.001 |  |  | 0.041 |
| 2013 | 1.24 (1.09, 1.42) | 0.001 |  |  |  |  |
| 2014 | 1.28 (1.12, 1.46) | <0.001 |  |  |  |  |
| 2015 | 1.22 (1.07, 1.40) | 0.003 |  |  |  |  |
| 2016 | 1.27 (1.12, 1.44) | <0.001 |  |  |  |  |
| 2017 | 1.17 (1.03, 1.33) | 0.018 |  | 1.18 (1.04, 1.34) | 0.010 |  |
| 2018 | 1.14 (1.01, 1.29) | 0.039 |  | 1.15 (1.02, 1.30) | 0.028 |  |
| 2019 | 1.06 (0.94, 1.20) | 0.347 |  | 1.07 (0.95, 1.21) | 0.266 |  |
| 2020 | 1.04 (0.92, 1.18) | 0.535 |  | 1.04 (0.91, 1.18) | 0.552 |  |
| 2021 | *ref* |  |  | *ref* |  |  |
| **Hospital beds** |  |  | 0.883 |  |  | 0.865 |
| >=500 |  |  |  |  |  |  |
| 150-499 | 1.02 (0.95, 1.09) | 0.628 |  | 1.02 (0.94, 1.11) | 0.657 |  |
| 0-150 | 1.01 (0.92, 1.10) | 0.890 |  | 1.03 (0.92, 1.15) | 0.627 |  |
| **Hospital ownership** |  |  | 0.001 |  |  | 0.027 |
| Government | 0.89 (0.80, 0.99) | 0.031 |  | 0.91 (0.79, 1.04) | 0.156 |  |
| Physician/Proprietary | *ref* |  |  | *ref* |  |  |
| Voluntary | 0.86 (0.80, 0.93) | <0.001 |  | 0.88 (0.80, 0.97) | 0.007 |  |
| **Region** |  |  | 0.240 |  |  | 0.036 |
| Northeast | 1.02 (0.93, 1.11) | 0.721 |  | 0.96 (0.86, 1.07) | 0.474 |  |
| South | 0.95 (0.88, 1.02) | 0.167 |  | 0.87 (0.79, 0.96) | 0.004 |  |
| Midwest | *ref* |  |  | *ref* |  |  |
| West | 0.93 (0.85, 1.02) | 0.131 |  | 0.92 (0.82, 1.02) | 0.122 |  |
| **Rurality** |  |  | 0.043 |  |  | 0.337 |
| Large Urban | *ref* |  |  | *ref* |  |  |
| Small Urban | 0.93 (0.87, 0.99) | 0.020 |  | 0.96 (0.89, 1.03) | 0.258 |  |
| Rural | 1.00 (0.91, 1.11) | 0.923 |  | 1.04 (0.91, 1.18) | 0.592 |  |
| **Resident-to-bed ratio** |  |  | 0.081 |  |  | 0.353 |
| No teaching | 0.90 (0.82, 1.00) | 0.046 |  | 0.95 (0.84, 1.07) | 0.422 |  |
| Minor teaching | 0.96 (0.86, 1.06) | 0.416 |  | 1.01 (0.89, 1.16) | 0.834 |  |
| Major teaching | *ref* |  |  | *ref* |  |  |
| **Dual Eligibility (yes)** |  |  |  | 1.34 (1.17, 1.53) | <0.001 | <0.001 |
| **C-statistic** | 0.67 |  |  | 0.66 |  |  |
| **ICC** | 0.02 |  |  | 0.01 |  |  |

**Supplemental Table 7: Mixed-effects models of PNBs on length of stay in THA patients**

|  | **Primary cohort** | | | **Secondary cohort  (including dual eligibility)** | | |
| --- | --- | --- | --- | --- | --- | --- |
| **Variables** | **OR (95% CI)** | **P-value OR** | **P-value variable** | **OR (95% CI)** | **P-value OR** | **P-value variable** |
| **Intercept** | 0.42 (0.29, 0.60) | <0.001 | <0.001 | 0.28 (0.18, 0.46) | <0.001 | <0.001 |
| **Peripheral nerve blocks** | 0.99 (0.89, 1.11) | 0.904 | 0.904 | 1.33 (1.14, 1.55) | <0.001 | <0.001 |
| **Age** |  |  | <0.001 |  |  | <0.001 |
| 65-69 | 0.60 (0.55, 0.66) | <0.001 |  | 0.59 (0.51, 0.68) | <0.001 |  |
| 70-74 | 0.75 (0.69, 0.81) | <0.001 |  | 0.75 (0.67, 0.85) | <0.001 |  |
| 75-79 | *ref* |  |  | *ref* |  |  |
| 80-84 | 1.32 (1.21, 1.44) | <0.001 |  | 1.32 (1.16, 1.51) | <0.001 |  |
| >84 | 2.09 (1.91, 2.29) | <0.001 |  | 2.12 (1.84, 2.43) | <0.001 |  |
| **Female** | 1.25 (1.18, 1.33) | <0.001 | <0.001 | 1.24 (1.13, 1.36) | <0.001 | <0.001 |
| **Inpatient (vs. outpatient)** |  |  |  |  |  |  |
| **Diagnosis non-osteoarthritis** | 4.97 (4.67, 5.30) | <0.001 | <0.001 | 9.25 (8.42, 10.16) | <0.001 | <0.001 |
| **Prior hospitalizations** |  |  | <0.001 |  |  | <0.001 |
| 0 | *ref* |  |  | *ref* |  |  |
| 1 | 1.37 (1.27, 1.48) | <0.001 |  | 1.33 (1.19, 1.49) | <0.001 |  |
| >=2 | 2.23 (2.00, 2.50) | <0.001 |  | 1.92 (1.63, 2.26) | <0.001 |  |
| **Deyo index** |  |  | <0.001 |  |  | <0.001 |
| 0 | 0.43 (0.40, 0.47) | <0.001 |  | 0.44 (0.39, 0.49) | <0.001 |  |
| 1 | 0.55 (0.51, 0.60) | <0.001 |  | 0.55 (0.49, 0.62) | <0.001 |  |
| 2 | 0.72 (0.66, 0.78) | <0.001 |  | 0.72 (0.64, 0.82) | <0.001 |  |
| >=3 | *ref* |  |  | *ref* |  |  |
| **No obesity** | 0.94 (0.88, 1.01) | 0.105 | 0.105 | 0.95 (0.86, 1.06) | 0.365 | 0.365 |
| **No abuse of non-opioids** | 0.75 (0.67, 0.85) | <0.001 | <0.001 | 0.82 (0.70, 0.97) | 0.017 | 0.017 |
| **No abuse of opioids** | 0.59 (0.46, 0.75) | <0.001 | <0.001 | 0.51 (0.39, 0.68) | <0.001 | <0.001 |
| **No smoking** | 1.00 (0.92, 1.10) | 0.957 | 0.957 | 1.19 (0.88, 1.60) | 0.252 | 0.252 |
| **Social Deprivation Index** |  |  | 0.009 |  |  | 0.173 |
| Q1 (most affluent) | *ref* |  |  | *ref* |  |  |
| Q2,3,4 | 1.03 (0.95, 1.13) | 0.441 |  | 1.08 (0.96, 1.23) | 0.204 |  |
| Q5 (least affluent) | 1.16 (1.05, 1.29) | 0.005 |  | 1.16 (0.99, 1.35) | 0.061 |  |
| **Race and Ethnicity** |  |  | 0.002 |  |  | 0.022 |
| White | *ref* |  |  | *ref* |  |  |
| Black | 1.19 (1.04, 1.37) | 0.012 |  | 1.31 (1.07, 1.61) | 0.010 |  |
| Asian, Hispanic, North American Native, other | 1.31 (1.07, 1.59) | 0.008 |  | 1.18 (0.89, 1.57) | 0.243 |  |
| **Year of surgery** |  |  | <0.001 |  |  | 0.001 |
| 2013 | 0.96 (0.83, 1.11) | 0.580 |  |  |  |  |
| 2014 | 0.78 (0.68, 0.91) | 0.002 |  |  |  |  |
| 2015 | 0.69 (0.60, 0.81) | <0.001 |  |  |  |  |
| 2016 | 0.98 (0.85, 1.14) | 0.823 |  |  |  |  |
| 2017 | 0.85 (0.73, 0.99) | 0.032 |  | 0.93 (0.80, 1.09) | 0.385 |  |
| 2018 | 0.76 (0.66, 0.89) | <0.001 |  | 0.83 (0.71, 0.97) | 0.021 |  |
| 2019 | 0.70 (0.60, 0.81) | <0.001 |  | 0.76 (0.65, 0.88) | <0.001 |  |
| 2020 | 0.86 (0.73, 1.00) | 0.053 |  | 0.88 (0.75, 1.04) | 0.130 |  |
| 2021 | *ref* |  |  | *ref* |  |  |
| **Hospital beds** |  |  | <0.001 |  |  | <0.001 |
| >=500 | *ref* |  |  | *ref* |  |  |
| 150-499 | 1.01 (0.91, 1.12) | 0.866 |  | 1.04 (0.92, 1.18) | 0.505 |  |
| 0-150 | 0.65 (0.56, 0.76) | <0.001 |  | 0.64 (0.52, 0.77) | <0.001 |  |
| **Hospital ownership** |  |  | <0.001 |  |  | 0.006 |
| Government | 1.13 (0.96, 1.34) | 0.137 |  | 1.04 (0.85, 1.28) | 0.694 |  |
| Physician/Proprietary | *ref* |  |  | *ref* |  |  |
| Voluntary | 0.87 (0.77, 0.98) | 0.023 |  | 0.83 (0.71, 0.97) | 0.017 |  |
| **Region** |  |  | <0.001 |  |  | 0.021 |
| Northeast | 1.32 (1.15, 1.52) | <0.001 |  | 1.17 (0.98, 1.39) | 0.075 |  |
| South | 1.36 (1.21, 1.53) | <0.001 |  | 1.10 (0.95, 1.28) | 0.189 |  |
| Midwest | *ref* |  |  | *ref* |  |  |
| West | 1.01 (0.88, 1.16) | 0.923 |  | 0.91 (0.76, 1.08) | 0.264 |  |
| **Rurality** |  |  | <0.001 |  |  | <0.001 |
| Large Urban | *ref* |  |  | *ref* |  |  |
| Small Urban | 0.88 (0.80, 0.97) | 0.011 |  | 0.91 (0.81, 1.02) | 0.100 |  |
| Rural | 1.50 (1.29, 1.75) | <0.001 |  | 1.40 (1.15, 1.71) | 0.001 |  |
| **Resident-to-bed ratio** |  |  | <0.001 |  |  | 0.001 |
| No teaching | 0.72 (0.62, 0.84) | <0.001 |  | 0.72 (0.61, 0.86) | <0.001 |  |
| Minor teaching | 0.82 (0.69, 0.96) | 0.017 |  | 0.85 (0.70, 1.03) | 0.097 |  |
| Major teaching | *ref* |  |  | *ref* |  |  |
| **Dual Eligibility (yes)** |  |  |  | 1.77 (1.51, 2.08) | <0.001 | <0.001 |
| **C-statistic** | 0.83 |  |  | 0.86 |  |  |
| **ICC** | 0.13 |  |  | 0.10 |  |  |

**Supplemental Table 8: Mixed-effects models of PNBs on length of stay in TKA patients**

|  | **Primary cohort** | | | **Secondary cohort  (including dual eligibility)** | | |
| --- | --- | --- | --- | --- | --- | --- |
| **Variables** | **OR (95% CI)** | **P-value OR** | **P-value variable** | **OR (95% CI)** | **P-value OR** | **P-value variable** |
| **Intercept** | 0.29 (0.20, 0.41) | <0.001 | <0.001 | 0.19 (0.12, 0.32) | <0.001 | <0.001 |
| **Peripheral nerve blocks** | 0.90 (0.86, 0.95) | <0.001 | <0.001 | 0.79 (0.73, 0.87) | <0.001 | <0.001 |
| **Age** |  |  | <0.001 |  |  | <0.001 |
| 65-69 | 0.69 (0.64, 0.74) | <0.001 |  | 0.64 (0.57, 0.72) | <0.001 |  |
| 70-74 | 0.79 (0.74, 0.84) | <0.001 |  | 0.79 (0.71, 0.87) | <0.001 |  |
| 75-79 | *ref* |  |  | *ref* |  |  |
| 80-84 | 1.30 (1.21, 1.40) | <0.001 |  | 1.26 (1.12, 1.42) | <0.001 |  |
| >84 | 1.67 (1.52, 1.83) | <0.001 |  | 1.80 (1.55, 2.09) | <0.001 |  |
| **Female** | 1.19 (1.13, 1.25) | <0.001 | <0.001 | 1.32 (1.21, 1.43) | <0.001 | <0.001 |
| **Inpatient (vs. outpatient)** |  |  |  |  |  |  |
| **Diagnosis non-osteoarthritis** | 1.51 (1.42, 1.60) | <0.001 | <0.001 | 3.01 (2.68, 3.39) | <0.001 | <0.001 |
| **Prior hospitalizations** |  |  | <0.001 |  |  | <0.001 |
| 0 | *ref* |  |  | *ref* |  |  |
| 1 | 1.28 (1.20, 1.37) | <0.001 |  | 1.36 (1.22, 1.52) | <0.001 |  |
| >=2 | 2.37 (2.13, 2.63) | <0.001 |  | 2.36 (2.01, 2.77) | <0.001 |  |
| **Deyo index** |  |  | <0.001 |  |  | <0.001 |
| 0 | 0.47 (0.44, 0.50) | <0.001 |  | 0.44 (0.39, 0.49) | <0.001 |  |
| 1 | 0.62 (0.58, 0.66) | <0.001 |  | 0.59 (0.53, 0.65) | <0.001 |  |
| 2 | 0.70 (0.66, 0.75) | <0.001 |  | 0.64 (0.57, 0.72) | <0.001 |  |
| >=3 | *ref* |  |  | *ref* |  |  |
| **No obesity** | 0.84 (0.79, 0.88) | <0.001 | <0.001 | 0.84 (0.78, 0.92) | <0.001 | <0.001 |
| **No abuse of non-opioids** | 0.87 (0.76, 0.98) | 0.027 | 0.027 | 0.81 (0.69, 0.97) | 0.019 | 0.019 |
| **No abuse of opioids** | 0.57 (0.45, 0.73) | <0.001 | <0.001 | 0.67 (0.51, 0.89) | 0.005 | 0.005 |
| **No smoking** | 0.97 (0.90, 1.05) | 0.440 | 0.440 | 1.20 (0.85, 1.69) | 0.300 | 0.300 |
| **Social Deprivation Index** |  |  | <0.001 |  |  | 0.021 |
| Q1 (most affluent) | *ref* |  |  | *ref* |  |  |
| Q2,3,4 | 1.13 (1.05, 1.21) | 0.002 |  | 1.14 (1.01, 1.28) | 0.032 |  |
| Q5 (least affluent) | 1.21 (1.11, 1.33) | <0.001 |  | 1.22 (1.06, 1.40) | 0.006 |  |
| **Race and Ethnicity** |  |  | <0.001 |  |  | 0.065 |
| White | *ref* |  |  | *ref* |  |  |
| Black | 1.24 (1.13, 1.37) | <0.001 |  | 1.20 (1.02, 1.40) | 0.027 |  |
| Asian, Hispanic, North American Native, other | 1.22 (1.08, 1.38) | 0.002 |  | 0.94 (0.77, 1.16) | 0.583 |  |
| **Year of surgery** |  |  | <0.001 |  |  | 0.001 |
| 2013 | 2.16 (1.87, 2.50) | <0.001 |  |  |  |  |
| 2014 | 1.68 (1.45, 1.94) | <0.001 |  |  |  |  |
| 2015 | 1.37 (1.18, 1.58) | <0.001 |  |  |  |  |
| 2016 | 1.24 (1.08, 1.44) | 0.003 |  |  |  |  |
| 2017 | 1.07 (0.93, 1.24) | 0.354 |  | 1.14 (0.98, 1.33) | 0.087 |  |
| 2018 | 0.92 (0.80, 1.07) | 0.299 |  | 0.98 (0.84, 1.15) | 0.807 |  |
| 2019 | 0.89 (0.76, 1.03) | 0.125 |  | 0.94 (0.81, 1.11) | 0.473 |  |
| 2020 | 0.93 (0.78, 1.09) | 0.361 |  | 0.92 (0.78, 1.10) | 0.367 |  |
| 2021 | *ref* |  |  | *ref* |  |  |
| **Hospital beds** |  |  | <0.001 |  |  | <0.001 |
| >=500 | *ref* |  |  | *ref* |  |  |
| 150-499 | 1.02 (0.93, 1.13) | 0.670 |  | 1.05 (0.93, 1.20) | 0.416 |  |
| 0-150 | 0.70 (0.61, 0.80) | <0.001 |  | 0.73 (0.61, 0.87) | 0.001 |  |
| **Hospital ownership** |  |  | <0.001 |  |  | 0.029 |
| Government | 1.26 (1.08, 1.46) | 0.003 |  | 1.13 (0.92, 1.38) | 0.235 |  |
| Physician/Proprietary | *ref* |  |  | *ref* |  |  |
| Voluntary | 0.92 (0.82, 1.03) | 0.131 |  | 0.90 (0.78, 1.04) | 0.170 |  |
| **Region** |  |  | <0.001 |  |  | 0.002 |
| Northeast | 1.24 (1.08, 1.41) | 0.002 |  | 1.20 (1.01, 1.42) | 0.038 |  |
| South | 1.25 (1.11, 1.39) | <0.001 |  | 1.19 (1.03, 1.38) | 0.019 |  |
| Midwest | *ref* |  |  | *ref* |  |  |
| West | 0.97 (0.85, 1.10) | 0.630 |  | 0.92 (0.77, 1.09) | 0.328 |  |
| **Rurality** |  |  | <0.001 |  |  | <0.001 |
| Large Urban | *ref* |  |  | *ref* |  |  |
| Small Urban | 0.87 (0.80, 0.96) | 0.004 |  | 0.87 (0.77, 0.98) | 0.024 |  |
| Rural | 1.40 (1.22, 1.60) | <0.001 |  | 1.39 (1.16, 1.67) | <0.001 |  |
| **Resident-to-bed ratio** |  |  | 0.001 |  |  | 0.002 |
| No teaching | 0.76 (0.66, 0.88) | <0.001 |  | 0.73 (0.61, 0.87) | 0.001 |  |
| Minor teaching | 0.81 (0.69, 0.95) | 0.010 |  | 0.81 (0.66, 0.99) | 0.037 |  |
| Major teaching | *ref* |  |  | *ref* |  |  |
| **Dual Eligibility (yes)** |  |  |  | 1.72 (1.50, 1.97) | <0.001 | <0.001 |
| **C-statistic** | 0.78 |  |  | 0.80 |  |  |
| **ICC** | 0.14 |  |  | 0.13 |  |  |

**Supplemental Table 9: Mixed-effects models of variables of interest on PNB use in THA patients**

|  | **Primary cohort: Model 1  (only SDI; main model in paper)** | | | **Primary cohort: Model 2  (including state-county ID)** | | | **Primary cohort: Model 3  (SDI and state-county ID)** | | |
| --- | --- | --- | --- | --- | --- | --- | --- | --- | --- |
| **Variables** | **OR (95% CI)** | **P-value OR** | **P-value variable** | **OR (95% CI)** | **P-value OR** | **P-value variable** | **OR (95% CI)** | **P-value OR** | **P-value variable** |
| **Intercept** | 0.21 (0.10, 0.43) | <0.001 | <0.001 | 0.20 (0.09, 0.41) | <0.001 | <0.001 | 0.20 (0.10, 0.43) | <0.001 | <0.001 |
| **Age** |  |  | 0.088 |  |  | 0.088 |  |  | 0.087 |
| 65-69 | 0.97 (0.86, 1.10) | 0.644 |  | 0.97 (0.86, 1.10) | 0.670 |  | 0.97 (0.86, 1.10) | 0.664 |  |
| 70-74 | 0.91 (0.82, 1.02) | 0.109 |  | 0.91 (0.82, 1.02) | 0.110 |  | 0.91 (0.82, 1.02) | 0.108 |  |
| 75-79 | *ref* |  |  |  |  |  |  |  |  |
| 80-84 | 0.86 (0.75, 0.98) | 0.027 |  | 0.86 (0.75, 0.99) | 0.030 |  | 0.86 (0.75, 0.98) | 0.029 |  |
| >84 | 0.84 (0.72, 0.99) | 0.038 |  | 0.84 (0.72, 0.99) | 0.038 |  | 0.84 (0.72, 0.99) | 0.037 |  |
| **Female** | 1.02 (0.93, 1.11) | 0.724 | 0.724 | 1.01 (0.93, 1.10) | 0.736 | 0.736 | 1.02 (0.93, 1.11) | 0.731 | 0.731 |
| **Inpatient (vs. outpatient)** | 1.28 (1.07, 1.53) | 0.007 | 0.007 | 1.28 (1.07, 1.54) | 0.007 | 0.007 | 1.28 (1.07, 1.54) | 0.007 | 0.007 |
| **Diagnosis non-osteoarthritis** | 0.64 (0.58, 0.72) | <0.001 | <0.001 | 0.64 (0.57, 0.72) | <0.001 | <0.001 | 0.64 (0.57, 0.72) | <0.001 | <0.001 |
| **Prior hospitalizations** |  |  | 0.015 |  |  | 0.014 |  |  | 0.014 |
| 0 | *ref* |  |  | 0.97 (0.86, 1.10) | 0.660 |  | 0.97 (0.86, 1.10) | 0.655 |  |
| 1 | 0.97 (0.86, 1.10) | 0.653 |  | 0.71 (0.57, 0.90) | 0.004 |  | 0.71 (0.57, 0.90) | 0.004 |  |
| >=2 | 0.72 (0.57, 0.90) | 0.004 |  |  |  |  |  |  |  |
| **Deyo index** |  |  | 0.640 |  |  | 0.634 |  |  | 0.629 |
| 0 | 0.96 (0.86, 1.08) | 0.525 |  | 0.96 (0.86, 1.08) | 0.530 |  | 0.96 (0.86, 1.08) | 0.525 |  |
| 1 | 1.00 (0.88, 1.12) | 0.953 |  | 1.00 (0.88, 1.12) | 0.957 |  | 1.00 (0.88, 1.13) | 0.960 |  |
| 2 | 0.93 (0.81, 1.06) | 0.249 |  | 0.93 (0.81, 1.06) | 0.246 |  | 0.92 (0.81, 1.05) | 0.244 |  |
| >=3 | *ref* |  |  |  |  |  |  |  |  |
| **No obesity** | 1.01 (0.91, 1.11) | 0.868 | 0.868 | 1.01 (0.91, 1.11) | 0.863 | 0.863 | 1.01 (0.91, 1.11) | 0.864 | 0.864 |
| **No abuse of non-opioids** | 0.96 (0.81, 1.14) | 0.654 | 0.654 | 0.96 (0.81, 1.14) | 0.659 | 0.659 | 0.96 (0.81, 1.14) | 0.657 | 0.657 |
| **No abuse of opioids** | 0.58 (0.40, 0.84) | 0.004 | 0.004 | 0.58 (0.40, 0.84) | 0.004 | 0.004 | 0.58 (0.40, 0.84) | 0.004 | 0.004 |
| **No smoking** | 1.07 (0.92, 1.25) | 0.380 | 0.380 | 1.07 (0.92, 1.25) | 0.365 | 0.365 | 1.07 (0.92, 1.25) | 0.370 | 0.370 |
| **Social Deprivation Index** |  |  | 0.549 |  |  |  |  |  | 0.639 |
| Q1 (most affluent) | *ref* |  |  |  |  |  |  |  |  |
| Q2,3,4 | 0.94 (0.82, 1.07) | 0.328 |  |  |  |  | 0.94 (0.82, 1.08) | 0.396 |  |
| Q5 (least affluent) | 0.98 (0.83, 1.16) | 0.842 |  |  |  |  | 0.98 (0.83, 1.17) | 0.850 |  |
| **Race and Ethnicity** |  |  | 0.488 |  |  | 0.478 |  |  | 0.482 |
| White | *ref* |  |  |  |  |  |  |  |  |
| Black | 0.95 (0.76, 1.17) | 0.620 |  | 0.95 (0.76, 1.18) | 0.630 |  | 0.95 (0.76, 1.18) | 0.625 |  |
| Asian, Hispanic, North American Native, other | 1.18 (0.87, 1.59) | 0.285 |  | 1.18 (0.88, 1.60) | 0.274 |  | 1.18 (0.87, 1.60) | 0.278 |  |
| **Year of surgery** |  |  | <0.001 |  |  | <0.001 |  |  | <0.001 |
| 2013 | 0.49 (0.39, 0.60) | <0.001 |  | 0.49 (0.39, 0.60) | <0.001 |  | 0.49 (0.39, 0.60) | <0.001 |  |
| 2014 | 0.53 (0.43, 0.65) | <0.001 |  | 0.53 (0.43, 0.65) | <0.001 |  | 0.53 (0.43, 0.65) | <0.001 |  |
| 2015 | 0.42 (0.34, 0.52) | <0.001 |  | 0.42 (0.34, 0.52) | <0.001 |  | 0.42 (0.34, 0.52) | <0.001 |  |
| 2016 | 0.34 (0.28, 0.42) | <0.001 |  | 0.34 (0.28, 0.41) | <0.001 |  | 0.34 (0.28, 0.41) | <0.001 |  |
| 2017 | 0.44 (0.36, 0.54) | <0.001 |  | 0.44 (0.36, 0.54) | <0.001 |  | 0.44 (0.36, 0.54) | <0.001 |  |
| 2018 | 0.52 (0.43, 0.63) | <0.001 |  | 0.52 (0.43, 0.63) | <0.001 |  | 0.52 (0.43, 0.63) | <0.001 |  |
| 2019 | 0.64 (0.53, 0.77) | <0.001 |  | 0.64 (0.53, 0.77) | <0.001 |  | 0.64 (0.53, 0.77) | <0.001 |  |
| 2020 | 0.67 (0.56, 0.79) | <0.001 |  | 0.66 (0.56, 0.79) | <0.001 |  | 0.66 (0.56, 0.79) | <0.001 |  |
| 2021 | *ref* |  |  |  |  |  |  |  |  |
| **Hospital beds** |  |  | 0.234 |  |  | 0.240 |  |  | 0.239 |
| >=500 | *ref* |  |  |  |  |  |  |  |  |
| 150-499 | 0.82 (0.60, 1.13) | 0.219 |  | 0.82 (0.60, 1.13) | 0.222 |  | 0.82 (0.60, 1.13) | 0.221 |  |
| 0-150 | 1.08 (0.73, 1.61) | 0.696 |  | 1.08 (0.73, 1.61) | 0.703 |  | 1.08 (0.73, 1.61) | 0.704 |  |
| **Hospital ownership** |  |  | <0.001 |  |  | <0.001 |  |  | <0.001 |
| Government | 0.71 (0.45, 1.13) | 0.152 |  | 0.71 (0.44, 1.13) | 0.148 |  | 0.71 (0.45, 1.13) | 0.150 |  |
| Physician/Proprietary | *ref* |  |  |  |  |  |  |  |  |
| Voluntary | 0.51 (0.36, 0.71) | <0.001 |  | 0.51 (0.36, 0.71) | <0.001 |  | 0.51 (0.36, 0.72) | <0.001 |  |
| **Region** |  |  | <0.001 |  |  | <0.001 |  |  | <0.001 |
| Northeast | 0.40 (0.26, 0.62) | <0.001 |  | 0.40 (0.26, 0.63) | <0.001 |  | 0.40 (0.26, 0.62) | <0.001 |  |
| South | 0.87 (0.62, 1.23) | 0.422 |  | 0.87 (0.62, 1.23) | 0.425 |  | 0.00 (0.00, 0.00) |  |  |
| Midwest | *ref* |  |  |  |  |  |  | 0.424 |  |
| West | 1.09 (0.73, 1.60) | 0.682 |  | 1.08 (0.73, 1.60) | 0.691 |  | 1.09 (0.73, 1.61) | 0.679 |  |
| **Rurality** |  |  | <0.001 |  |  | <0.001 |  |  | <0.001 |
| Large Urban | *ref* |  |  |  |  |  |  |  |  |
| Small Urban | 0.54 (0.40, 0.72) | <0.001 |  | 0.53 (0.40, 0.71) | <0.001 |  | 0.54 (0.40, 0.72) | <0.001 |  |
| Rural | 0.74 (0.49, 1.12) | 0.159 |  | 0.74 (0.49, 1.11) | 0.146 |  | 0.74 (0.49, 1.12) | 0.155 |  |
| **Resident-to-bed ratio** |  |  | 0.035 |  |  | 0.034 |  |  | 0.034 |
| No teaching | 0.55 (0.35, 0.86) | 0.010 |  | 0.55 (0.34, 0.86) | 0.009 |  | 0.55 (0.34, 0.86) | 0.009 |  |
| Minor teaching | 0.62 (0.37, 1.04) | 0.071 |  | 0.62 (0.37, 1.03) | 0.067 |  | 0.62 (0.37, 1.04) | 0.068 |  |
| Major teaching | *ref* |  |  |  |  |  |  |  |  |
| **C-statistic** | 0.94 |  |  | 0.94 |  |  | 0.94 |  |  |
| **ICC** | 0.67 |  |  | 0.67 |  |  | 0.67 |  |  |
| **Difference in log-likelihood** | *ref* |  |  | *vs. model 1: 0.47* |  |  | *vs. model 2: 0.44* |  |  |

**Supplemental Table 10: Mixed-effects models of variables of interest on PNB use in TKA patients**

|  | **Primary cohort: Model 1  (only SDI; main model in paper)** | | | **Primary cohort: Model 2  (including state-county ID)** | | | **Primary cohort: Model 3  (SDI and state-county ID)** | | |
| --- | --- | --- | --- | --- | --- | --- | --- | --- | --- |
| **Variables** | **OR (95% CI)** | **P-value OR** | **P-value variable** | **OR (95% CI)** | **P-value OR** | **P-value variable** | **OR (95% CI)** | **P-value OR** | **P-value variable** |
| **Intercept** | 6.92 (4.57, 10.46) | <0.001 | <0.001 | 6.90 (4.57, 10.42) | <0.001 | <0.001 | 6.92 (4.57, 10.48) | <0.001 | <0.001 |
| **Age** |  |  | <0.001 |  |  | <0.001 |  |  | <0.001 |
| 65-69 | 0.84 (0.80, 0.88) | <0.001 |  | 0.84 (0.80, 0.88) | <0.001 |  | 0.84 (0.80, 0.88) | <0.001 |  |
| 70-74 | 0.90 (0.86, 0.94) | <0.001 |  | 0.90 (0.86, 0.94) | <0.001 |  | 0.90 (0.86, 0.94) | <0.001 |  |
| 75-79 | *ref* |  |  |  |  |  |  |  |  |
| 80-84 | 0.97 (0.91, 1.02) | 0.246 |  | 0.97 (0.91, 1.03) | 0.264 |  | 0.97 (0.91, 1.03) | 0.263 |  |
| >84 | 0.81 (0.75, 0.88) | <0.001 |  | 0.81 (0.75, 0.88) | <0.001 |  | 0.81 (0.75, 0.88) | <0.001 |  |
| **Female** | 0.95 (0.92, 0.99) | 0.008 | 0.008 | 0.95 (0.92, 0.99) | 0.007 | 0.007 | 0.95 (0.92, 0.99) | 0.008 | 0.008 |
| **Inpatient (vs. outpatient)** | 0.71 (0.67, 0.76) | <0.001 | <0.001 | 0.71 (0.67, 0.76) | <0.001 | <0.001 | 0.71 (0.67, 0.76) | <0.001 | <0.001 |
| **Diagnosis non-osteoarthritis** | 0.35 (0.34, 0.37) | <0.001 | <0.001 | 0.35 (0.34, 0.37) | <0.001 | <0.001 | 0.35 (0.34, 0.37) | <0.001 | <0.001 |
| **Prior hospitalizations** |  |  | <0.001 |  |  | <0.001 |  |  | <0.001 |
| 0 | *ref* |  |  | 0.97 (0.92, 1.03) | 0.327 |  | 0.97 (0.92, 1.03) | 0.327 |  |
| 1 | 0.97 (0.92, 1.03) | 0.328 |  | 0.76 (0.68, 0.84) | <0.001 |  | 0.76 (0.68, 0.84) | <0.001 |  |
| >=2 | 0.76 (0.68, 0.84) | <0.001 |  |  |  |  |  |  |  |
| **Deyo index** |  |  | 0.015 |  |  | 0.017 |  |  | 0.017 |
| 0 | 0.95 (0.90, 0.99) | 0.019 |  | 0.95 (0.90, 0.99) | 0.019 |  | 0.95 (0.90, 0.99) | 0.019 |  |
| 1 | 1.01 (0.96, 1.06) | 0.705 |  | 1.01 (0.96, 1.06) | 0.713 |  | 1.01 (0.96, 1.06) | 0.714 |  |
| 2 | 0.96 (0.91, 1.02) | 0.178 |  | 0.96 (0.91, 1.02) | 0.189 |  | 0.96 (0.91, 1.02) | 0.189 |  |
| >=3 | *ref* |  |  |  |  |  |  |  |  |
| **No obesity** | 0.98 (0.95, 1.02) | 0.344 | 0.344 | 0.98 (0.94, 1.02) | 0.319 | 0.319 | 0.98 (0.94, 1.02) | 0.319 | 0.319 |
| **No abuse of non-opioids** | 0.98 (0.89, 1.07) | 0.595 | 0.595 | 0.98 (0.89, 1.07) | 0.592 | 0.592 | 0.98 (0.89, 1.07) | 0.588 | 0.588 |
| **No abuse of opioids** | 1.04 (0.85, 1.28) | 0.671 | 0.671 | 1.04 (0.85, 1.27) | 0.720 | 0.720 | 1.04 (0.85, 1.27) | 0.717 | 0.717 |
| **No smoking** | 0.88 (0.83, 0.94) | <0.001 | <0.001 | 0.88 (0.83, 0.94) | <0.001 | <0.001 | 0.88 (0.83, 0.94) | <0.001 | <0.001 |
| **Social Deprivation Index** |  |  | 0.322 |  |  |  |  |  | 0.570 |
| Q1 (most affluent) | *ref* |  |  |  |  |  |  |  |  |
| Q2,3,4 | 1.00 (0.95, 1.06) | 0.942 |  |  |  |  | 1.00 (0.94, 1.07) | 0.893 |  |
| Q5 (least affluent) | 0.96 (0.89, 1.03) | 0.248 |  |  |  |  | 0.97 (0.89, 1.05) | 0.456 |  |
| **Race and Ethnicity** |  |  | 0.049 |  |  | 0.036 |  |  | 0.043 |
| White | *ref* |  |  |  |  |  |  |  |  |
| Black | 0.91 (0.84, 0.99) | 0.029 |  | 0.91 (0.84, 0.99) | 0.021 |  | 0.91 (0.84, 0.99) | 0.026 |  |
| Asian, Hispanic, North American Native, other | 0.94 (0.85, 1.04) | 0.213 |  | 0.94 (0.85, 1.03) | 0.196 |  | 0.94 (0.85, 1.04) | 0.207 |  |
| **Year of surgery** |  |  | <0.001 |  |  | <0.001 |  |  | <0.001 |
| 2013 | 0.83 (0.76, 0.91) | <0.001 |  | 0.84 (0.76, 0.92) | <0.001 |  | 0.84 (0.76, 0.92) | <0.001 |  |
| 2014 | 0.62 (0.56, 0.68) | <0.001 |  | 0.62 (0.56, 0.68) | <0.001 |  | 0.62 (0.56, 0.68) | <0.001 |  |
| 2015 | 0.55 (0.50, 0.60) | <0.001 |  | 0.55 (0.50, 0.60) | <0.001 |  | 0.55 (0.50, 0.60) | <0.001 |  |
| 2016 | 0.47 (0.43, 0.51) | <0.001 |  | 0.47 (0.43, 0.51) | <0.001 |  | 0.47 (0.43, 0.51) | <0.001 |  |
| 2017 | 0.68 (0.63, 0.75) | <0.001 |  | 0.69 (0.63, 0.75) | <0.001 |  | 0.69 (0.63, 0.75) | <0.001 |  |
| 2018 | 0.80 (0.74, 0.87) | <0.001 |  | 0.80 (0.74, 0.87) | <0.001 |  | 0.80 (0.74, 0.87) | <0.001 |  |
| 2019 | 0.85 (0.79, 0.93) | <0.001 |  | 0.85 (0.79, 0.93) | <0.001 |  | 0.85 (0.79, 0.93) | <0.001 |  |
| 2020 | 0.91 (0.83, 0.99) | 0.024 |  | 0.91 (0.83, 0.99) | 0.024 |  | 0.91 (0.83, 0.99) | 0.023 |  |
| 2021 | *ref* |  |  |  |  |  |  |  |  |
| **Hospital beds** |  |  | 0.137 |  |  | 0.133 |  |  | 0.138 |
| >=500 | *ref* |  |  |  |  |  |  |  |  |
| 150-499 | 0.85 (0.70, 1.02) | 0.075 |  | 0.85 (0.70, 1.02) | 0.074 |  | 0.85 (0.70, 1.02) | 0.076 |  |
| 0-150 | 0.98 (0.79, 1.23) | 0.876 |  | 0.98 (0.79, 1.23) | 0.886 |  | 0.98 (0.79, 1.23) | 0.880 |  |
| **Hospital ownership** |  |  | 0.727 |  |  | 0.723 |  |  | 0.728 |
| Government | 0.94 (0.72, 1.23) | 0.661 |  | 0.94 (0.72, 1.23) | 0.657 |  | 0.94 (0.72, 1.23) | 0.650 |  |
| Physician/Proprietary | *ref* |  |  |  |  |  |  |  |  |
| Voluntary | 1.03 (0.85, 1.26) | 0.732 |  | 1.03 (0.85, 1.26) | 0.730 |  | 1.03 (0.85, 1.25) | 0.746 |  |
| **Region** |  |  | 0.005 |  |  | 0.005 |  |  | 0.007 |
| Northeast | 0.74 (0.58, 0.94) | 0.012 |  | 0.74 (0.58, 0.94) | 0.015 |  | 0.74 (0.58, 0.94) | 0.015 |  |
| South | 0.74 (0.61, 0.90) | 0.003 |  | 0.73 (0.60, 0.89) | 0.002 |  | 0.00 (0.00, 0.00) |  |  |
| Midwest | *ref* |  |  |  |  |  |  | 0.003 |  |
| West | 0.71 (0.56, 0.89) | 0.003 |  | 0.71 (0.57, 0.89) | 0.004 |  | 0.72 (0.57, 0.90) | 0.004 |  |
| **Rurality** |  |  | <0.001 |  |  | <0.001 |  |  | <0.001 |
| Large Urban | *ref* |  |  |  |  |  |  |  |  |
| Small Urban | 0.80 (0.67, 0.94) | 0.007 |  | 0.80 (0.67, 0.94) | 0.008 |  | 0.80 (0.67, 0.94) | 0.008 |  |
| Rural | 0.50 (0.40, 0.63) | <0.001 |  | 0.50 (0.40, 0.63) | <0.001 |  | 0.50 (0.40, 0.63) | <0.001 |  |
| **Resident-to-bed ratio** |  |  | 0.425 |  |  | 0.424 |  |  | 0.422 |
| No teaching | 0.96 (0.73, 1.26) | 0.780 |  | 0.97 (0.74, 1.28) | 0.847 |  | 0.97 (0.74, 1.27) | 0.828 |  |
| Minor teaching | 0.84 (0.62, 1.15) | 0.282 |  | 0.85 (0.63, 1.16) | 0.306 |  | 0.85 (0.62, 1.16) | 0.298 |  |
| Major teaching | *ref* |  |  |  |  |  |  |  |  |
| **C-statistic** | 0.87 |  |  | 0.87 |  |  | 0.87 |  |  |
| **ICC** | 0.49 |  |  | 0.49 |  |  | 0.49 |  |  |
| **Difference in log likelihood** | *ref* |  |  | *vs. model 1: 10.28* |  |  | *vs. model 2: 0.55* |  |  |

**Supplemental Table 11: Mixed-effects models of variables of interest including dual eligibility on PNB use in THA and TKA patients**

|  | **THA** | | | **TKA** | | |
| --- | --- | --- | --- | --- | --- | --- |
|  | **Secondary cohort  (including dual eligibility)** | | | **Secondary cohort  (including dual eligibility)** | | |
| **Variables** | **OR (95% CI)** | **P-value OR** | **P-value variable** | **OR (95% CI)** | **P-value OR** | **P-value variable** |
| **Intercept** | 0.09 (0.03, 0.24) | <0.001 | <0.001 | 7.37 (4.28, 12.68) | <0.001 | <0.001 |
| **Age** |  |  | 0.124 |  |  | <0.001 |
| 65-69 | 0.97 (0.82, 1.14) | 0.719 |  | 0.83 (0.78, 0.89) | <0.001 |  |
| 70-74 | 0.84 (0.72, 0.98) | 0.025 |  | 0.89 (0.84, 0.95) | 0.001 |  |
| 75-79 | *ref* |  |  | *ref* |  |  |
| 80-84 | 0.84 (0.70, 1.02) | 0.076 |  | 0.98 (0.90, 1.07) | 0.724 |  |
| >84 | 0.92 (0.73, 1.15) | 0.456 |  | 0.92 (0.82, 1.04) | 0.202 |  |
| **Female** | 1.01 (0.90, 1.14) | 0.872 | 0.872 | 0.94 (0.89, 0.99) | 0.014 | 0.014 |
| **Inpatient (vs. outpatient)** | 1.30 (1.06, 1.59) | 0.011 | 0.011 | 0.79 (0.74, 0.85) | <0.001 | <0.001 |
| **Diagnosis non-osteoarthritis** | 0.48 (0.40, 0.57) | <0.001 | <0.001 | 0.08 (0.07, 0.09) | <0.001 | <0.001 |
| **Prior hospitalizations** |  |  | 0.207 |  |  | 0.097 |
| 0 | *ref* |  |  | *ref* |  |  |
| 1 | 0.86 (0.72, 1.03) | 0.108 |  | 0.97 (0.89, 1.06) | 0.517 |  |
| >=2 | 0.86 (0.63, 1.17) | 0.335 |  | 0.84 (0.72, 0.99) | 0.035 |  |
| **Deyo index** |  |  | 0.524 |  |  | 0.002 |
| 0 | 0.96 (0.82, 1.12) | 0.567 |  | 0.88 (0.83, 0.95) | <0.001 |  |
| 1 | 1.05 (0.89, 1.24) | 0.548 |  | 0.96 (0.90, 1.03) | 0.301 |  |
| 2 | 0.93 (0.78, 1.11) | 0.430 |  | 0.94 (0.87, 1.02) | 0.151 |  |
| >=3 | *ref* |  |  | *ref* |  |  |
| **No obesity** | 1.03 (0.90, 1.17) | 0.693 | 0.693 | 0.95 (0.90, 1.01) | 0.080 | 0.080 |
| **No abuse of non-opioids** | 0.88 (0.70, 1.10) | 0.267 | 0.267 | 0.92 (0.81, 1.04) | 0.163 | 0.163 |
| **No abuse of opioids** | 0.58 (0.37, 0.92) | 0.020 | 0.020 | 0.95 (0.75, 1.20) | 0.658 | 0.658 |
| **No smoking** | 1.48 (0.94, 2.33) | 0.092 | 0.092 | 1.06 (0.83, 1.35) | 0.633 | 0.633 |
| **Social Deprivation Index** |  |  | 0.985 |  |  | 0.377 |
| Q1 (most affluent) | *ref* |  |  | *ref* |  |  |
| Q2,3,4 | 0.99 (0.82, 1.18) | 0.872 |  | 1.03 (0.95, 1.11) | 0.511 |  |
| Q5 (least affluent) | 0.99 (0.80, 1.24) | 0.964 |  | 0.97 (0.88, 1.07) | 0.559 |  |
| **Race and Ethnicity** |  |  | 0.579 |  |  | 0.047 |
| White | *ref* |  |  | *ref* |  |  |
| Black | 0.99 (0.73, 1.33) | 0.937 |  | 0.86 (0.77, 0.97) | 0.016 |  |
| Asian, Hispanic, North American Native, other | 1.24 (0.83, 1.85) | 0.300 |  | 0.95 (0.82, 1.10) | 0.467 |  |
| **Year of surgery** |  |  | <0.001 |  |  | <0.001 |
| 2013 |  |  |  |  |  |  |
| 2014 |  |  |  |  |  |  |
| 2015 |  |  |  |  |  |  |
| 2016 |  |  |  |  |  |  |
| 2017 | 0.40 (0.33, 0.50) | <0.001 |  | 0.60 (0.54, 0.65) | <0.001 |  |
| 2018 | 0.47 (0.38, 0.57) | <0.001 |  | 0.73 (0.66, 0.79) | <0.001 |  |
| 2019 | 0.57 (0.47, 0.70) | <0.001 |  | 0.79 (0.73, 0.87) | <0.001 |  |
| 2020 | 0.62 (0.51, 0.74) | <0.001 |  | 0.89 (0.82, 0.98) | 0.013 |  |
| 2021 | *ref* |  |  | *ref* |  |  |
| **Hospital beds** |  |  | 0.135 |  |  | 0.049 |
| >=500 | *ref* |  |  | *ref* |  |  |
| 150-499 | 0.91 (0.59, 1.39) | 0.653 |  | 0.76 (0.60, 0.96) | 0.020 |  |
| 0-150 | 1.46 (0.87, 2.45) | 0.153 |  | 0.92 (0.70, 1.22) | 0.580 |  |
| **Hospital ownership** |  |  | 0.002 |  |  | 0.517 |
| Government | 0.53 (0.29, 0.98) | 0.044 |  | 0.84 (0.60, 1.18) | 0.323 |  |
| Physician/Proprietary | *ref* |  |  | *ref* |  |  |
| Voluntary | 0.45 (0.29, 0.71) | <0.001 |  | 1.00 (0.78, 1.27) | 0.983 |  |
| **Region** |  |  | 0.001 |  |  | 0.107 |
| Northeast | 0.35 (0.20, 0.62) | <0.001 |  | 0.75 (0.56, 1.01) | 0.060 |  |
| South | 1.03 (0.66, 1.62) | 0.886 |  | 0.77 (0.60, 0.99) | 0.039 |  |
| Midwest | *ref* |  |  | *ref* |  |  |
| West | 1.09 (0.65, 1.83) | 0.733 |  | 0.91 (0.69, 1.22) | 0.541 |  |
| **Rurality** |  |  | 0.001 |  |  | 0.001 |
| Large Urban | *ref* |  |  | *ref* |  |  |
| Small Urban | 0.51 (0.35, 0.74) | <0.001 |  | 0.82 (0.67, 1.02) | 0.070 |  |
| Rural | 0.95 (0.56, 1.62) | 0.843 |  | 0.57 (0.43, 0.76) | <0.001 |  |
| **Resident-to-bed ratio** |  |  | 0.064 |  |  | 0.913 |
| No teaching | 0.49 (0.27, 0.90) | 0.020 |  | 1.01 (0.72, 1.42) | 0.968 |  |
| Minor teaching | 0.61 (0.30, 1.21) | 0.154 |  | 0.95 (0.65, 1.40) | 0.796 |  |
| Major teaching | *ref* |  |  | *ref* |  |  |
| **Dual Eligibility (yes)** | 0.80 (0.60, 1.05) | 0.108 | 0.108 | 0.94 (0.84, 1.05) | 0.281 | 0.281 |
| **C-statistic** | 0.96 |  | <0.001 | 0.90 |  |  |
| **ICC** | 0.77 |  |  | 0.58 |  |  |
